# Combining Cyclic Triimidazo Triazine Core With Ethynyl‐*N*‐Methyl‐Pyridinium Groups for Targeting G‐Quadruplex Structures

**DOI:** 10.1002/ardp.70037

**Published:** 2025-07-02

**Authors:** Chiara Platella, Stefano Di Ciolo, Andrea Criscuolo, Daniele Malpicci, Rosa Gaglione, Angela Arciello, Domenica Musumeci, Elena Lucenti, Elena Cariati, Daniela Montesarchio, Clelia Giannini

**Affiliations:** ^1^ Department of Chemical Sciences University of Naples Federico II Napoli Italy; ^2^ Department of Chemistry University of Milan Milano Italy; ^3^ Institute of Biostructures and Bioimaging (IBB) ‐ CNR Napoli Italy; ^4^ Institute of Chemical Sciences and Technologies “Giulio Natta” (SCITEC) of CNR Milano Italy

**Keywords:** antiproliferative activity, biophysical characterization, chemical synthesis, cyclic triimidazo triazine derivatives, G‐quadruplex ligands

## Abstract

The synthesis and characterization of a mini‐library of cyclic triimidazo triazine (**TT**) derivatives functionalized with one, two, or three ethynyl‐*N*‐methyl‐pyridinium moieties are reported here. These compounds were designed with the aim of targeting cancer‐related DNA G‐quadruplex structures. The newly synthesized compounds were tested for their ability to bind G‐quadruplexes from both telomeric and oncogene promoter sequences using an affinity chromatography‐based assay, spectroscopic and electrophoretic techniques, as well as molecular docking analysis. The obtained results demonstrated the effective capacity of the investigated compounds to specifically recognize the selected G‐quadruplex models, with their **TT** cores targeting the outer G‐quartets and their positively charged *N*‐methyl‐pyridinium groups interacting with the top edge of G‐quadruplex grooves. Notably, the trisubstituted cyclic triimidazole compounds showed higher stabilizing properties than the related disubstituted derivatives, which in turn were stronger binders than their monosubstituted analogs. However, the mono‐ and disubstituted derivatives showed higher G‐quadruplex versus duplex recognition selectivity compared with the trisubstituted ones. Altogether, the biophysical experiments, also in agreement with the biological assays, underlined the advantage of introducing an alkyne linker between the triimidazole core and the methylpyridinium group, proving to be beneficial to increase both the stabilizing effects on the G‐quadruplexes and the anticancer activity compared with the analogs of the same family lacking the alkyne linker.

## Graphical abstract

A mini‐library of cyclic triimidazo triazine derivatives functionalized with one, two, or three ethynyl‐*N*‐methyl‐pyridinium moieties was synthesized, characterized for the interaction with cancer‐related G‐quadruplexes, and tested in antiproliferative activity screenings on human cancer cells. The alkyne linker between the triimidazole core and the *N*‐methylpyridinium group proved to be beneficial for both the stabilization of the G‐quadruplexes and the anticancer activity.

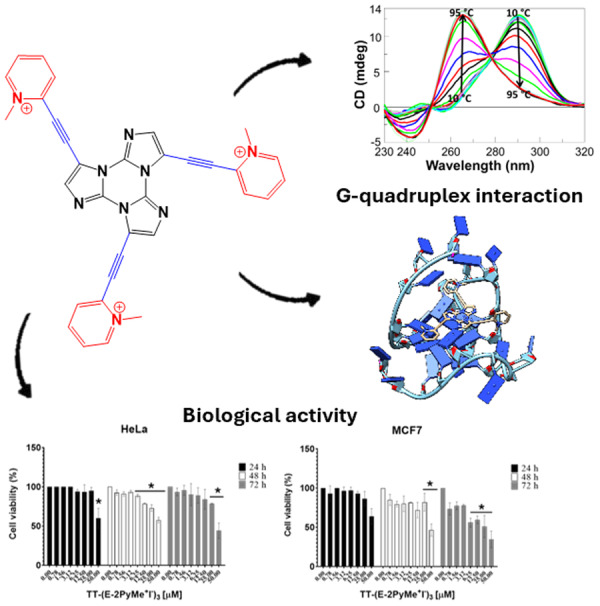

## 1 Introduction

G‐quadruplexes (G4) are noncanonical four‐stranded nucleic acid secondary structures formed by G‐rich sequences through stacking of two or more guanine quartets (G‐quartets) [1]. It has been found that they can play important roles in the regulation of genomic processes in various regions of the genome, including telomeres and promoter regions of oncogenes, such as telomere maintenance, replication, recombination, and regulation of oncogene expression [1].

Due to their role in key biological processes, G‐quadruplexes are being explored as potential therapeutic targets, particularly for the development of targeted cancer treatment [1, 2, 3]. Therefore, the development of ligands able to interact with these structures and regulate their function is a hot research topic in anticancer strategies. To date, a significant number of ligands targeting G‐quadruplexes have been investigated, and many of them have been deposited in the G4 Ligands Database 2.1 [4]. Most of the well‐known G‐quadruplex ligands share common structural features, that is, an aromatic core, which allows π − π stacking interactions with planar G‐quartets, and one or more positively charged moieties, providing strong interactions with DNA or RNA backbone phosphate groups in grooves and loops [4, 5, 6, 7, 8]. Despite the large number of already known compounds able to recognize and stabilize G‐quadruplexes, still very few of them have entered clinical trials [9]. Therefore, it is of the utmost importance to reinforce the research on the design and synthesis of new, effective G‐quadruplex‐selective ligands.

In recent years, some of us have prepared and characterized triimidazo[1,2‐*a*:1′,2′‐*c*:1″,2″‐*e*][1,3,5]triazine or cyclic triimidazole (hereafter **TT**) derivatives with a special focus on their photophysical behavior [10]. **TTs** are featured by a compact, nitrogen‐rich scaffold with *C*_*3h*_ symmetry, whose peculiar photoluminescent properties have been implemented by functionalization with various substituents. A number of **TTs** have revealed intriguing emissive properties, spanning from anti‐Kasha luminescence to room temperature multiple phosphorescence in the solid state [11, 12]. In addition, triazine functionalities are frequently used in the biological field [13, 14] due to their ability to act as central scaffolds for further modifications or as mimetics of purine moieties [15]. Among **TT** derivatives, 3‐(pyren‐1‐yl)‐triimidazo[1,2‐*a*:1′,2′‐*c*:1″,2″‐*e*][1,3,5]triazine, named **TTPyr**, has been successfully tested for potential bioimaging applications, including cellular and bacterial imaging [16]. Remarkably, **TTPyr** has been shown to form stable aggregates in water, which proved to stain both eukaryotic and prokaryotic cell lines, being able to easily cross cellular membranes and localize at cytoplasm level.

Moreover, since the **TT** scaffold shares certain common structural features with known G‐quadruplex‐binding small molecules [17] (i.e., the rigid flat structure typical of polycyclic heteroaromatic compounds) and can be rather easily functionalized with one or multiple pendant groups, endowed with various positively charged (or chargeable) moieties, we have recently investigated its potential also in this field [18]. More in detail, **TT** analogs functionalized with one, two, or three pyridine moieties attached to the central rings system via pyridine C‐2 or C‐4 were synthesized and then *N*‐methylated, obtaining derivatives carrying a positive charge differently located with respect to the central scaffold. Comparison between the para‐ and ortho‐substituted pyridine families revealed that the para‐substituted ones had better affinity for the target G‐quadruplex, highlighting the importance of the right distance of the positive charge from the central **TT** core [18].

Based on these results, in the present work, we performed a detailed investigation on brand new putative G‐quadruplex ligands based on the **TT** scaffold in which an ethynyl moiety has been inserted between the triazine system and the methylated pyridines. This functional group has been chosen not only to provide a longer linker to connect the positively charged moieties and the aromatic core but also to extend the π‐electron system responsible for the π–π interactions with the G‐quartets, thus in principle enabling better G‐quadruplex stabilization. The synthetic strategy for preparing ethynylpyridine derivatives of **TT** involved a Sonogashira coupling between the bromine‐functionalized **TT** derivative (**TT‐Br**, **TT‐Br**_**2**_, and **TT‐Br**_**3**_) and 2‐ or 4‐ethynylpyridine. The pyridine moieties were then *N*‐methylated, thus obtaining positively charged derivatives with the charge placed at different distances from the central core.

The interaction of the newly synthesized molecules with telomeric and oncogenic DNA G‐quadruplexes, as well as with control duplex models, was investigated by multiple biophysical techniques in a combined approach. In detail, the G4‐CPG (G‐quadruplex on Controlled Pore Glass) assay [19, 20]—a cheap and easy affinity chromatography‐based method developed by some of us—was exploited to assess the capacity of the examined synthetic compounds to interact with G‐quadruplexes, as well as their G‐quadruplex versus duplex selectivity. Then, these compounds were tested in solution for their ability to bind and/or thermally stabilize the target G‐quadruplex structures by circular dichroism (CD), using a DNA duplex model as control. Additionally, native polyacrylamide gel electrophoresis (PAGE) experiments were carried out to obtain information on the molecularity of the formed complexes. Further insight into the binding mode of the compounds to the G‐quadruplex and duplex models was obtained by molecular docking studies. Moreover, the here‐investigated compounds were tested for their antiproliferative activity on human cancer cell lines.

## 2 Results and Discussion

### 2.1 Synthesis of the Methylated Ethynylpyridine TT Derivatives

One, two, or three ethynylpyridine moieties were linked to the **TT** scaffold starting from the corresponding **TT**‐brominated derivatives. Subsequent methylation by reaction with CH_3_I produced the target compounds endowed with an aromatic, planar central core and one, two, or three positively charged polar tails.

**Scheme 1:**
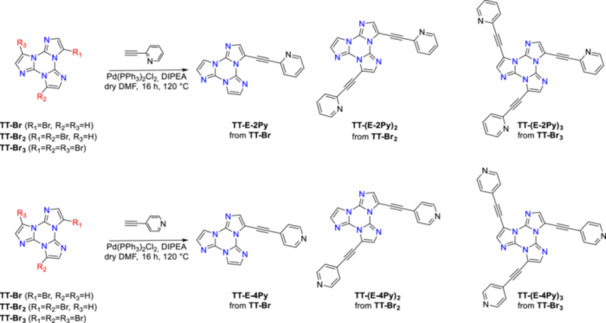
Synthetic route for the preparation of **TT‐E‐2Py**, **TT‐(E‐2Py)**_**2**_, **TT‐(E‐2Py)**_**3**_, **TT‐E‐4Py**, **TT‐(E‐4Py)**_**2**_ and **TT‐(E‐4Py)**_**3**_.

In detail, the brominated derivatives, that is, 3‐bromotriimidazo[1,2‐*a*:1′,2′‐*c*:1″,2″‐*e*][1,3,5]triazine (**TT‐Br**), 3,7‐dibromotriimidazo[1,2‐*a*:1′,2′‐*c*:1″,2″‐*e*][1,3,5]triazine (**TT‐Br**_**2**_), and 3,7,11‐tribromotriimidazo[1,2‐*a*:1′,2′‐*c*:1″,2″‐*e*][1,3,5]triazine (**TT‐Br**_**3**_) were prepared as previously reported [11, 21]. The ethynylpyridine derivatives **TT‐E‐2Py**, **TT‐(E‐2Py)**_**2**_, and **TT‐(E‐2Py)**_**3**_, as well as **TT‐E‐4Py**, **TT‐(E‐4Py)**_**2**_, and **TT‐(E‐4Py)**_**3**_, were obtained by Sonogashira coupling of **TT‐Br**_**1**_, **TT‐Br**_**2**_, and **TT‐Br**_**3**_ with 2‐ethynylpyridine or 4‐ethynylpyridine, respectively (Scheme 1). The crude reaction products were purified using standard chromatographic techniques, followed by precipitation, which provided the pure target compounds, then characterized by ^1^H and ^13^C NMR spectroscopy and mass spectrometry.

**Scheme 2:**
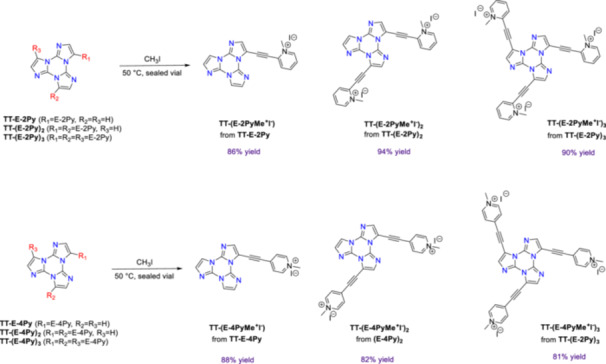
Synthetic route to the methylated ethynylpyridine **TT** derivatives.

In all the obtained compounds, the nitrogen atoms of the pyridine rings were methylated by reaction with CH_3_I (Scheme 2). NMR data provided experimental evidence that the methylation occurred selectively at the level of the pyridine moieties (see Section 4). Indeed, the nitrogen atoms of the **TT** core react only with very strong methylating agents, such as methyl trifluoromethanesulfonate, and thus were not affected by the CH_3_I treatment [22].

The newly synthesized compounds were evaluated for their ability to interact with G‐quadruplex targets, as well as their selectivity to recognize G‐quadruplex over duplex DNA, and the data were compared with those of the previously reported analogs **TT‐(4PyMe** ^**+**^ **I** ^**−**^ **)**_**2**_ and **TT‐(4PyMe** ^**+**^ **I** ^**−**^ **)**_**3**_ (Supporting Information S2: Chart S1) [18], here used as references to evaluate the role of the alkyne linker to connect the methylated pyridine to the **TT** scaffold.

### 2.2 Binding Assessment of the TT Derivatives to the G‐Quadruplex Targets and to a Control Duplex by the G4‐CPG Assay

First, to evaluate the ability of the newly prepared compounds to interact with the selected G‐quadruplex targets as well as their selectivity to recognize G‐quadruplexes over duplex DNA, the affinity chromatography‐based assay, named G‐quadruplex on Controlled Pore Glass (G4‐CPG) assay, was exploited [19, 20, 23]. This method consists of incubating the potential ligands with CPG beads functionalized with G‐quadruplex‐ or duplex‐forming oligonucleotides. The fractions recovered upon elution with proper releasing solutions are analyzed by simple UV measurements, and the percentages of each ligand bound to the targets or the control can be thus easily quantified [19, 20, 23].

As G‐quadruplex targets, we selected both a telomeric and an oncogenic G‐quadruplex, and particularly the telomeric sequence tel26 d[(TTAGGG)_4_TT], able to fold into a hybrid G‐quadruplex [24], and the oncogenic sequence c‐myc d(TGGGGAGGGTGGGGAGGGTGGGGAAGGTGGGGA), able to fold into parallel G‐quadruplex(es) [25, 26, 27]. As control, we used a unimolecular duplex‐forming DNA sequence, named ds27, consisting of two self‐complementary tracts, each containing the Dickerson sequence d(CGCGAATTCGCG), that is, one of the most studied models for B‐DNA [28], connected by a TTT loop.

The new **TT** derivatives were first tested on nude CPG beads to exclude the presence of unspecific binding (Table 1) and subsequently on the G‐quadruplex‐ and duplex‐functionalized beads. Altogether, the tested compounds showed strong binding interactions (Table 1) with the G‐quadruplex targets tel26 and c‐myc (percentages of bound ligand comprised between 65% and 100%). Notably, the trisubstituted compounds **TT‐(E‐2PyMe** ^**+**^ **I** ^**−**^ **)**_**3**_, **TT‐(E‐4PyMe** ^**+**^ **I** ^**−**^ **)**_**3**_, and **TT‐(4PyMe** ^**+**^ **I** ^**−**^ **)**_**3**_ showed higher affinities than the related disubstituted compounds **TT‐(E‐2PyMe** ^**+**^ **I** ^**−**^ **)**_**2**_, **TT‐(E‐4PyMe** ^**+**^ **I** ^**−**^ **)**_**2**_, and **TT‐(4PyMe** ^**+**^ **I** ^**−**^ **)**_**2**_, which in turn showed higher affinities compared with the monosubstituted compounds **TT‐(E‐2PyMe** ^**+**^ **I** ^**−**^ **)** and **TT‐(E‐4PyMe** ^**+**^ **I** ^**−**^ **)** (Table 1).

**Table 1:** Summary of the binding assay data obtained for the here‐investigated compounds as determined by the G4‐CPG assay. The amounts of bound ligand are calculated as a difference between the initially loaded amount of ligand and the unbound ligand, recovered by the washing solution (50 mM KCl, 10% DMSO, 10% CH_3_CH_2_OH), and are expressed as a percentage of the quantity initially loaded on each support. Errors associated with the reported percentages are typically within ±2%. The selectivity index is the ratio between the percentages of ligand bound to the indicated supports.

	Bound ligand (%)				Selectivity index		
Compound	Nude CPG	CPG‐tel26	CPG‐c‐myc	CPG‐ds27	CPG‐tel26/CPG‐ds27	CPG‐c‐myc/CPG‐ds27	CPG‐c‐myc/CPG‐tel26
**TT‐(E‐2PyMe** ^**+**^ **I** ^**−**^ **)**	0	72	85	10	7.2	8.5	1.2
**TT‐(E‐2PyMe** ^**+**^ **I** ^**−**^ **)**_**2**_	0	74	84	39	1.9	2.2	1.1
**TT‐(E‐2PyMe** ^**+**^ **I** ^**−**^ **)**_**3**_	4	89	99	56	1.6	1.8	1.1
**TT‐(E‐4PyMe** ^**+**^ **I** ^**−**^ **)**	3	66	88	15	4.4	5.9	1.3
**TT‐(E‐4PyMe** ^**+**^ **I** ^**−**^ **)**_**2**_	0	79	100	40	2.0	2.5	1.3
**TT‐(E‐4PyMe** ^**+**^ **I** ^**−**^ **)**_**3**_	0	75	90	50	1.5	1.8	1.2
**TT‐(4PyMe** ^**+**^ **I** ^**−**^ **)**_**2**_	6	65	83	21	3.1	3.9	1.3
**TT‐(4PyMe** ^**+**^ **I** ^**−**^ **)**_**3**_	2	79	100	52	1.5	1.9	1.3

From the ratio between the percentage of ligand bound to the G‐quadruplex targets and the ligand bound to the control duplex, a selectivity index was determined (Table 1), proving that monosubstituted compounds had higher G‐quadruplex over duplex selectivity compared with disubstituted, which in turn were more selective than trisubstituted compounds. On the basis of the ratio between the percentages of ligand bound to the parallel G‐quadruplex c‐myc and the ligand bound to the hybrid G‐quadruplex tel26 (Table 1), the analyzed compounds revealed a slight preference for the parallel G‐quadruplex c‐myc.

### 2.3 Solution Studies on the Interaction of the TT Derivatives With the G‐Quadruplex Targets and a Control Duplex by CD

The ability of the here‐synthesized compounds to interact with the G‐quadruplex targets was also evaluated in solution by CD, using a duplex DNA as a control. The oligonucleotide models for the telomeric G‐quadruplex and the control duplex were the same ones used in the G4‐CPG assay, that is, tel26 and the self‐complementary Dickerson dodecamer named ds12. On the other hand, considering that the c‐myc sequence d(TGGGGAGGGTGGGGAGGGTGGGGAAGGTGGGGA) is characterized by the coexistence of multiple parallel G‐quadruplexes in equilibrium [25, 26, 27] and thus does not represent a good model for semi‐quantitative solution studies, one of its shorter variants of sequence d(TGAGGGTGGGTAGGGTGGGTAA), named pu22, was here selected for the biophysical studies, being featured by a single major G‐quadruplex conformation [29].

The G‐quadruplex targets, as well as the control duplex, were prepared by annealing the oligonucleotide solutions at 2 μM concentration, in 20 mM KCl, 5 mM potassium phosphate buffer (pH 7) for tel26 and ds12 or 10 mM Tris–HCl buffer (pH 7) for pu22. Under these conditions, in agreement with the literature, the structures adopted by tel26, pu22, and ds12 were a hybrid 2‐type G‐quadruplex [30]—also defined as hybrid‐3 according to a recently adopted G‐quadruplex classification [31]—a parallel G‐quadruplex [32] and a B‐DNA duplex [33, 34], respectively, as confirmed by the CD profiles observed for each free oligonucleotide (Supporting Information S2: Figure S1, left panels).

Then, the three oligonucleotides were titrated with increasing amounts of each compound, and the corresponding CD spectra were recorded after each addition (Supporting Information S2: Figures S2‐S9, left panels).

**Figure 1:**
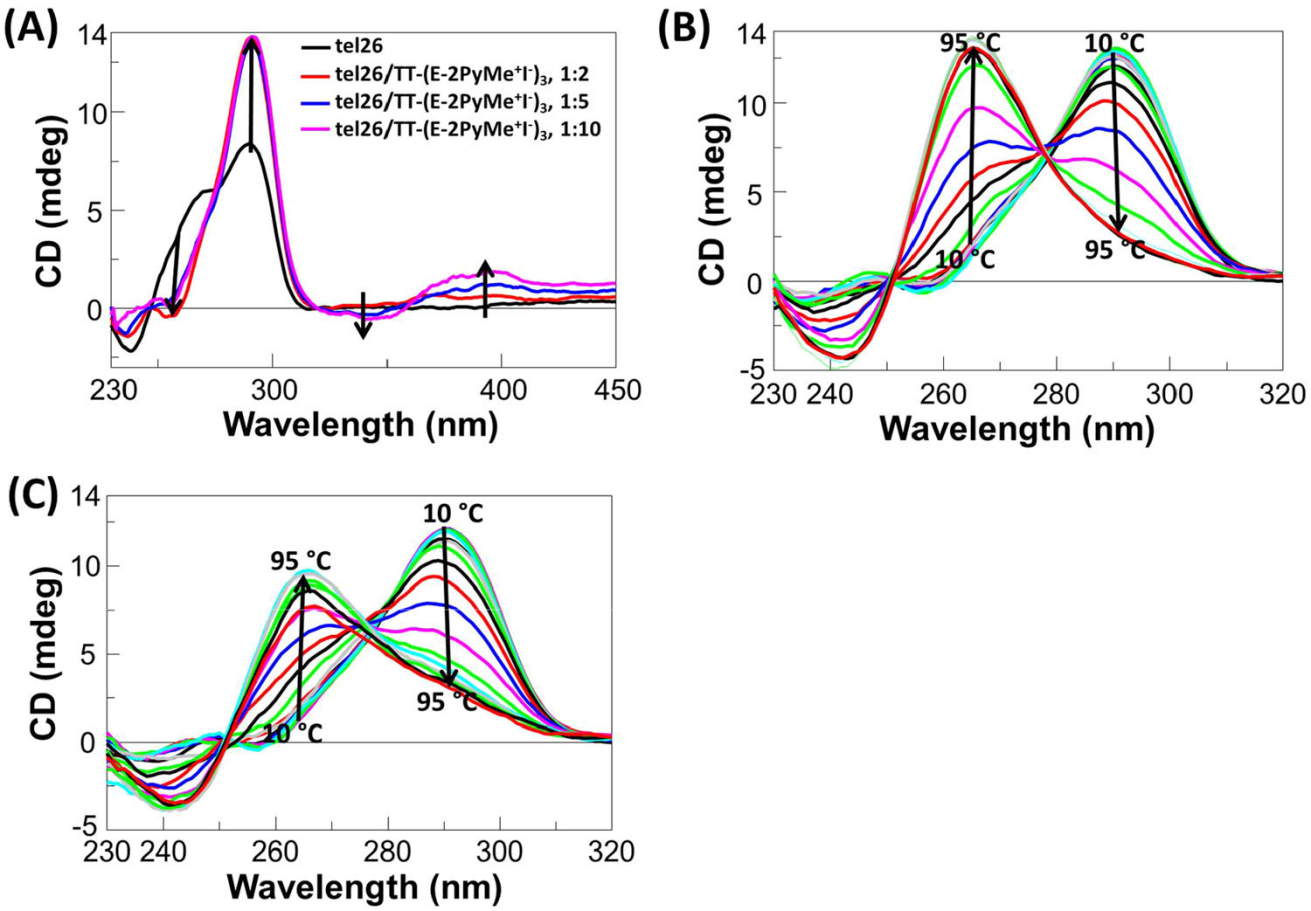
(A) CD spectra of 2 μM solutions of tel26 in 20 mM KCl, 5 mM potassium phosphate buffer (pH 7) in the presence of increasing amounts (up to 10 equivalents) of **TT‐(E‐2PyMe** ^**+**^ **I** ^**–**^ **)**_**3**_. Arrows indicate the variation of CD bands on increasing ligand concentration. (B) and (C) CD spectra of 2 μM solutions of tel26 in 20 mM KCl, 5 mM potassium phosphate buffer in the presence of 2 equivalents of **TT‐(E‐2PyMe** ^**+**^ **I** ^**–**^ **)**_**3**_ (B) and **TT‐(E‐4PyMe** ^**+**^ **I** ^**–**^ **)**_**3**_ (C) at different temperatures. Arrows indicate the variation of CD bands on increasing the temperature from 10°C to 95°C.

CD titration experiments of tel26 showed a dose‐dependent increase of the 290 nm band along with a reduction of the 270 nm shoulder intensity (Supporting Information S2: Figures S2‐S9, panels A), with more marked effects on going from the mono‐ to the trisubstituted **TT** derivative. Interestingly, the appearance of an additional shoulder at ca. 250 nm was observed in the case of tel26 titration with the disubstituted compounds **TT‐(E‐2PyMe** ^**+**^ **I** ^**–**^ **)**_**2**_, **TT‐(E‐4PyMe** ^**+**^ **I** ^**–**^ **)**_**2**_, and **TT‐(4PyMe** ^**+**^ **I** ^**–**^ **)**_**2**_ and the trisubstituted compounds **TT‐(E‐2PyMe** ^**+**^ **I** ^**–**^ **)**_**3**_, **TT‐(E‐4PyMe** ^**+**^ **I** ^**–**^ **)**_**3**_, and **TT‐(4PyMe** ^**+**^ **I** ^**–**^ **)**_**3**_, suggesting a conformational conversion of the tel26 G‐quadruplex from hybrid‐2 to hybrid‐1 topology upon binding [35]. Moreover, in the case of the titration of tel26 with **TT‐(E‐2PyMe** ^**+**^ **I** ^**–**^ **)**_**3**_ (Figure 1A), both a positive and a negative induced CD bands were observed, further confirming the binding of this ligand to the target G‐quadruplex [36].

As far as the CD experiments with pu22 are concerned, a dose‐dependent increase of the intensity of both the positive 263 and negative 240 nm bands was observed upon titration with the investigated compounds (Supporting Information S2: Figures S2‐S9, panels B). Also in this case, increasingly more marked effects were observed in the G‐quadruplex structure—corresponding to a higher degree of parallel G‐quadruplex structuration—on increasing the degree of substitution of the **TT** core.

Notably, only very small changes in the CD profile of the control duplex were observed upon interaction with the disubstituted compounds **TT‐(E‐2PyMe** ^**+**^ **I** ^**–**^ **)**_**2**_ and **TT‐(E‐4PyMe** ^**+**^ **I** ^**–**^ **)**_**2**_ and for the trisubstituted compounds **TT‐(E‐2PyMe** ^**+**^ **I** ^**–**^ **)**_**3**_, **TT‐(E‐4PyMe** ^**+**^ **I** ^**–**^ **)**_**3**_, and **TT‐(4PyMe** ^**+**^ **I** ^**–**^ **)**_**3**_ (Supporting Information S2: Figures S2‐S9, panels C), whereas no relevant effects were found upon treatment with the monosubstituted compounds **TT‐(E‐2PyMe** ^**+**^ **I** ^**–**^ **)** and **TT‐(E‐4PyMe** ^**+**^ **I** ^**–**^ **)**.

Moreover, the ability of all compounds to affect the thermal stability of the G‐quadruplex targets and the control duplex was assessed by CD melting experiments (Supporting Information S2: Figures S2‐S9, right panels) comparing the related melting temperature (*T*_m_) values of the free oligonucleotides with those of the oligonucleotide/ligand systems. *T*_m_ values of 43°C, 27°C, and 65°C were found for free tel26, pu22, and ds12, respectively (Supporting Information S2: Figure S1, right panels). The *T*_m_ and Δ*T*_m_ values for the oligonucleotide/ligand systems are summarized in Table 2. All compounds were able to strongly stabilize tel26 and pu22 G‐quadruplexes. In detail, Δ*T*_m_ values ranged between +3 and +23°C for tel26 and between +8 and +63°C for pu22. Notably, in the case of pu22 incubated with **TT‐(E‐2PyMe** ^**+**^ **I** ^**−**^ **)**_**2**_, **TT‐(E‐2PyMe** ^**+**^ **I** ^**−**^ **)**_**3**_, and **TT‐(E‐4PyMe** ^**+**^ **I** ^**−**^ **)**_**3**_, two inflection points were observed in each melting curve, suggesting the presence of two different species in solution, both more stable than the free pu22 (Supporting Information S2: Figures S3, S4 and S7). Based on inspection of the CD spectra recorded at different temperatures during the melting experiments, which were all featured by the typical profile of parallel G‐quadruplexes (Supporting Information S2: Figure S10), it could be hypothesized that these ligands are able to bind pu22 with different binding modes, resulting in a different stabilization of pu22 parallel G‐quadruplex(es). On the other hand, no major effect was detected on the stability of the control duplex for all compounds (Δ*T*_m_ = –2 or +4°C), with the exception of **TT‐(E‐2PyMe** ^**+**^ **I** ^**−**^ **)**_**3**_ and **TT‐(E‐4PyMe** ^**+**^ **I** ^**−**^ **)**_**3**_ (Δ*T*_m_ = +15°C). Interestingly, by analysis of the CD spectra changes on increasing the temperature, differently from what was observed for free tel26 and pu22 (Supporting Information S2: Figure S11) as well as for all the other here‐investigated ligands (Supporting Information S2: Figure S12), **TT‐(E‐2PyMe** ^**+**^ **I** ^**−**^ **)**_**3**_ and **TT‐(E‐4PyMe** ^**+**^ **I** ^**−**^ **)**_**3**_ were found able to induce on tel26 a conformational switch from hybrid‐2 to a parallel G‐quadruplex topology (Figure 1B,C and Supporting Information S2: Figure S12), as well as to stabilize the parallel fold of pu22 so that even at 95°C it was not completely unfolded (Supporting Information S2: Figure S13). In detail, at ca. 70°C, the hybrid G‐quadruplex topology of tel26 was almost completely unfolded, whereas the parallel topology became predominant and remained stable even at 95°C.

**Table 2:** Melting temperature (*T*_m_) values of tel26, pu22, and ds12 in the presence of the investigated compounds (2 molar equivalents) as measured by CD melting experiments in 20 mM KCl, 5 mM potassium phosphate buffer (pH 7) for tel26 and ds12 or 10 mM Tris–HCl buffer (pH 7) for pu22. Δ*T*_m_ = *T*_m_(DNA/ligand, 1:2) – *T*_m_(free DNA).

	tel26		pu22		ds12		
Compound	*T*_m_ ± 1 (°C)	Δ*T*_m_ (°C)	*T*_m_ ± 1 (°C)	Δ*T*_m_ (°C)	*T*_m_ ± 1 (°C)		Δ*T*_m_ (°C)
**TT‐(E‐2PyMe** ^**+**^ **I** ^**−**^ **)**	46	+3	37	+10	63		−2
**TT‐(E‐2PyMe** ^**+**^ **I** ^**−**^ **)**_**2**_	63	+20	39/79	+12/ + 57	68		+3
**TT‐(E‐2PyMe** ^**+**^ **I** ^**−**^ **)**_**3**_	65	+22	43/90	+16/ + 63	80		+15
**TT‐(E‐4PyMe** ^**+**^ **I** ^**−**^ **)**	46	+3	35	+8	66		+1
**TT‐(E‐4PyMe** ^**+**^ **I** ^**−**^ **)**_**2**_	61	+18	72	+45	69		+4
**TT‐(E‐4PyMe** ^**+**^ **I** ^**−**^ **)**_**3**_	66	+23	46/84	+19/ + 57	80		+15
**TT‐(4PyMe** ^**+**^ **I** ^**−**^ **)**_**2**_	53	+10	45	+18	66		+1
**TT‐(4PyMe** ^**+**^ **I** ^**−**^ **)**_**3**_	61	+18	62	+35	66		+1

Taken together, these results demonstrated the ability of the here‐investigated compounds to interact with the telomeric and oncogenic G‐quadruplex models, in full agreement with the G4‐CPG assay data, showing strong stabilizing effects on both tel26 and pu22 G‐quadruplexes but not on the control duplex.

Notably, the trisubstituted compounds **TT‐(E‐2PyMe** ^**+**^ **I** ^**−**^ **)**_**3**_, **TT‐(E‐4PyMe** ^**+**^ **I** ^**−**^ **)**_**3**_, and **TT‐(4PyMe** ^**+**^ **I** ^**−**^ **)**_**3**_ showed higher stabilizing properties than the related disubstituted compounds **TT‐(E‐2PyMe** ^**+**^ **I** ^**−**^ **)**_**2**_, **TT‐(E‐4PyMe** ^**+**^ **I** ^**−**^ **)**_**2**_, and **TT‐(4PyMe** ^**+**^ **I** ^**−**^ **)**_**2**_, which in turn were stronger stabilizers than the monosubstituted compounds **TT‐(E‐2PyMe** ^**+**^ **I** ^**−**^ **)** and **TT‐(E‐4PyMe** ^**+**^ **I** ^**−**^ **)**. Overall, the monosubstituted and disubstituted compounds proved to have higher G‐quadruplex over duplex selectivity compared with the trisubstituted compounds. Finally, the obtained results suggested that the introduction of an alkyne linker between the triazine **TT** core and the methylpyridinium pendant group was beneficial to increase the stabilizing effects on the G‐quadruplex targets (Table 2).

### 2.4 PAGE Experiments

To obtain further information on the binding of the investigated compounds to tel26 and pu22 G‐quadruplexes, and particularly on the molecularity of the formed complexes, native PAGE experiments were carried out, using the ds12 duplex as a control.

**Figure 2:**
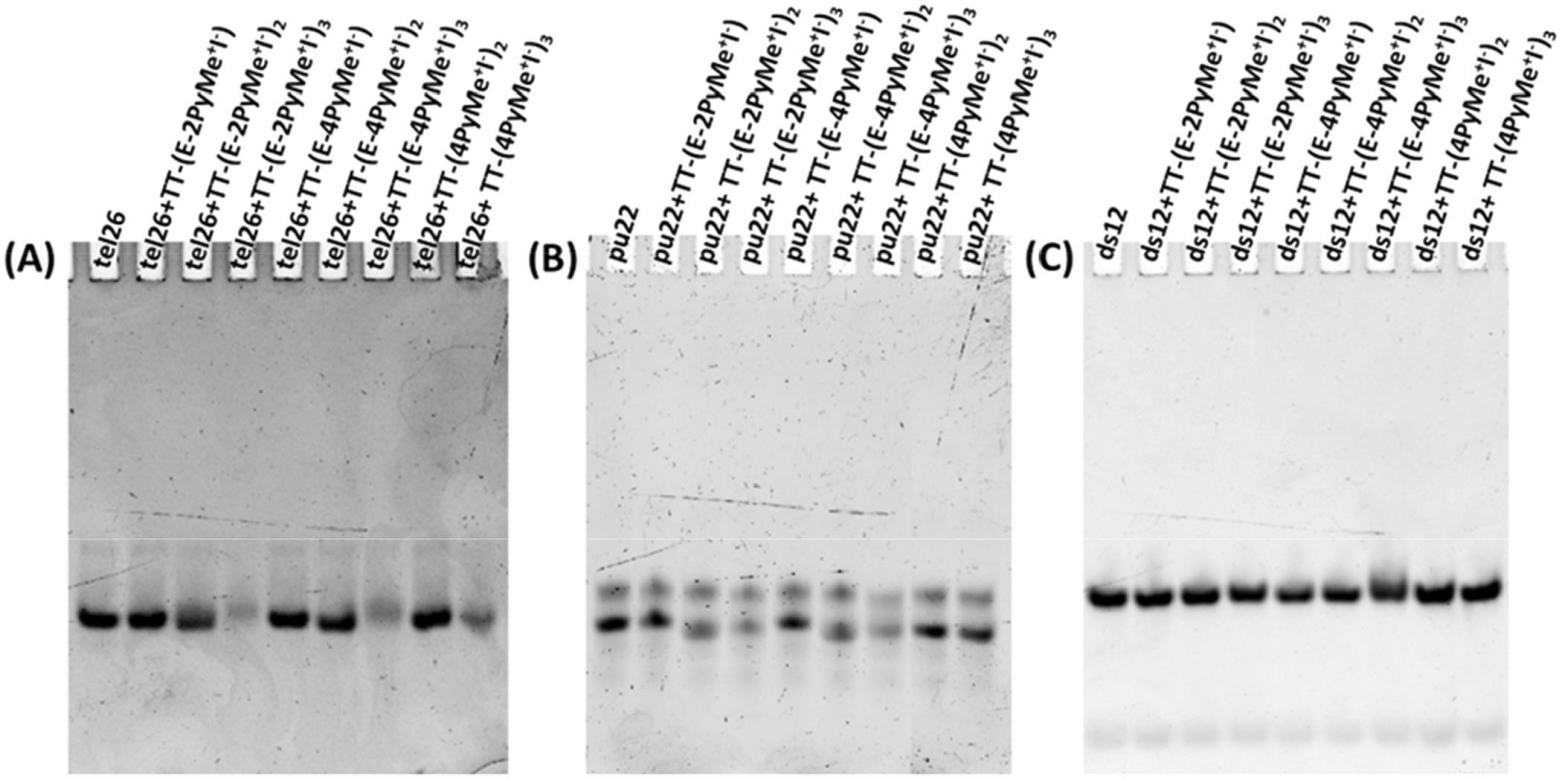
Native PAGE experiments: pu22 samples were loaded at 2 μM concentration in 20 mM KCl, 5 mM potassium phosphate buffer (pH 7) for tel26 and ds12, or 10 mM Tris–HCl buffer (pH 7) for pu22, in the absence and presence of different ligands (2 molar equivalents) and analyzed by 20% native PAGE using Tris‐Borate‐EDTA (TBE 1x), supplemented with 25 mM KCl for tel26 and ds12, as running buffer (2.5 h, 100 V, r.t.). Gels were visualized by GelGreen staining.

Native PAGE experiments were performed by analyzing tel26, pu22, and ds12 samples at 2 µM concentration in 20 mM KCl, 5 mM potassium phosphate buffer (pH 7) for tel26 and ds12 or 10 mM Tris–HCl buffer (pH 7) for pu22, mixed with 2 molar equivalents of each ligand, thus using the same conditions exploited for the CD experiments (Figure 2).

The free tel26 and ds12 showed a main intense band, corresponding to the 26‐mer hybrid G‐quadruplex formed by tel26 and the 24‐mer bimolecular duplex formed by ds12, respectively. In the case of ds12, also a faster band was observed, which can be attributed to a small amount of the related 12‐mer single strand, dissociated on the gel. On the other hand, pu22 showed two bands with slightly different mobility: the main and faster band is attributable to the parallel G‐quadruplex folding of pu22, while the less intense and slower band could probably be due to a small amount of oligonucleotide partially unfolded under the gel experimental conditions. Indeed, it can be excluded that the slower band is due to dimeric or higher order G‐quadruplexes of pu22, since the migration of this band is much faster than the band corresponding to the dimeric G‐quadruplex structure of the telomeric sequence formed by the oligonucleotide d[AGGG(TTAGGG)_7_] (tel46), here used as control (Supporting Information S2: Figure S14).

Notably, no mobility or intensity changes were observed for any of the ds12 bands related to the incubation of the duplex with each of the ligands with respect to the free duplex band (Figure 2C). On the other hand, slight variations on the band mobility were observed for both tel26 and pu22 incubated with the disubstituted compounds **TT‐(E‐2PyMe** ^**+**^ **I** ^**−**^ **)**_**2**_ and **TT‐(E‐4PyMe** ^**+**^ **I** ^**−**^ **)**_**2**_ or with the trisubstituted compounds **TT‐(E‐2PyMe** ^**+**^ **I** ^**−**^ **)**_**3**_, **TT‐(E‐4PyMe** ^**+**^ **I** ^**−**^ **)**_**3**_, and **TT‐(4PyMe** ^**+**^ **I** ^**−**^ **)**_**3**_ (Figure 2A,B).

Interestingly, a reduction in the intensity of tel26 G‐quadruplex was observed incubating the oligonucleotide sequence with the trisubstituted compounds **TT‐(E‐2PyMe** ^**+**^ **I** ^**−**^ **)**_**3**_, **TT‐(E‐4PyMe** ^**+**^ **I** ^**−**^ **)**_**3**_, and **TT‐(4PyMe** ^**+**^ **I** ^**−**^ **)**_**3**_ (Figure 2A), while for pu22 G‐quadruplex bands this effect was observed incubating the oligonucleotide sequence with the di‐ and trisubstituted ethynylpyridine **TT** compounds **TT‐(E‐2PyMe** ^**+**^ **I** ^**−**^ **)**_**2**_, **TT‐(E‐2PyMe** ^**+**^ **I** ^**−**^ **)**_**3**_, **TT‐(E‐4PyMe** ^**+**^ **I** ^**−**^ **)**_**2**_, and **TT‐(E‐4PyMe** ^**+**^ **I** ^**−**^ **)**_**3**_ (Figure 2B). On the other hand, no relevant differences in the band intensity were observed for both tel26 and pu22 with the monosubstituted **TT** compounds, nor, for pu22, with the previously reported analogs without the ethynyl linker, **TT‐(4PyMe** ^**+**^ **I** ^**−**^ **)**_**2**_ and **TT‐(4PyMe** ^**+**^ **I** ^**−**^ **)**_**3**_. A possible explanation of these results can be found in the lower accessibility of GelGreen staining to its binding sites on the G‐quadruplex if already occupied by the ligands, thus further proving that the binding of the di‐ and/or trisubstituted ethynylpyridine **TT** compounds to tel26 and/or pu22 G‐quadruplexes was stronger than in the case of **TT‐(E‐2PyMe** ^**+**^ **I** ^**−**^ **)**, **TT‐(E‐4PyMe** ^**+**^ **I** ^**−**^ **)**, **TT‐(4PyMe** ^**+**^ **I** ^**−**^ **)**_**2**_, and **TT‐(4PyMe** ^**+**^ **I** ^**−**^ **)**_**3**_.

Overall, the gel electrophoresis analysis proved that the here‐tested ligands do not promote the formation of higher order species starting from the monomeric hybrid tel26 and parallel pu22 G‐quadruplexes, nor do they produce any effects on the control ds12 duplex. Thus, the high Δ*T*_m_ values observed for pu22 cannot be associated with the formation of high‐stability higher order species but are due to the different stabilization induced by the ligands on the monomeric parallel folding(s) of pu22. In agreement with the G4‐CPG assay and CD experiments, the di‐ and/or trisubstituted ethynylpyridinium **TT** derivatives interacted with tel26 and/or pu22 G‐quadruplexes with a higher affinity compared with the corresponding monosubstituted compounds as well as to the previously reported di‐ and trisubstituted pyridinium analogs without the ethynyl linker.

### 2.5 Biological Assays

Preliminary evaluation of the antiproliferative activity of the compounds here studied was carried out by MTT (3‐(4,5‐dimethylthiazol‐2‐yl)‐ 2,5‐diphenyltetrazolium bromide) assays on human HeLa adenocarcinoma cells and human MCF7 breast cancer cells. Generally, all the new compounds showed dose‐dependent cytotoxic effects upon incubation with the tested cell lines (Supporting Information S2: Figures S15–S16), contrarily to the previously reported control analogs **TT‐(4PyMe** ^**+**^ **I** ^**−**^ **)**_**2**_ and **TT‐(4PyMe** ^**+**^ **I** ^**−**^ **)**_**3**_, which did not show any relevant cytotoxic activity in the explored range of concentrations (0–50 μM) and incubation times (24, 48, and 72 h).

Overall, **TT‐(E‐2PyMe** ^**+**^ **I** ^**−**^ **)**, **TT‐(E‐2PyMe** ^**+**^ **I** ^**−**^ **)**_**2**_, **TT‐(E‐2PyMe** ^**+**^ **I** ^**−**^ **)**_**3**_, and **TT‐(E‐4PyMe** ^**+**^ **I** ^**−**^ **)**_**3**_ proved to be the most active species in the series (Table 3), showing the lowest IC_50_ values compared with all the other analogs for both the tested cell lines (IC_50_ values in the micromolar range determined at 72 h incubation). Biological assays, in agreement with biophysical experiments, underlined—by comparison with previously studied **TT‐(4PyMe** ^**+**^ **I** ^**−**^ **)**_**2**_ and **TT‐(4PyMe** ^**+**^ **I** ^**−**^ **)**_**3**_—the importance of the introduction of an alkyne linker between the triimidazole core and the methylpyridinium pendant group to increase the antiproliferative activity of this class of compounds.

**Table 3:** IC_50_ values were established by testing increasing concentrations of each compound in the 0–50 µM range for 72 h on HeLa and MCF7 cancer cells by MTT assays.

	IC_50_ (µM)	
Compound	HeLa	MCF7
**TT‐(E‐2PyMe** ^**+**^ **I** ^**−**^ **)**	41	> 50
**TT‐(E‐2PyMe** ^**+**^ **I** ^**−**^ **)**_**2**_	> 50	38
**TT‐(E‐2PyMe** ^**+**^ **I** ^**−**^ **)**_**3**_	47	29
**TT‐(E‐4PyMe** ^**+**^ **I** ^**−**^ **)**	> 50	> 50
**TT‐(E‐4PyMe** ^**+**^ **I** ^**−**^ **)**_**2**_	> 50	> 50
**TT‐(E‐4PyMe** ^**+**^ **I** ^**−**^ **)**_**3**_	32	50
**TT‐(4PyMe** ^**+**^ **I** ^**−**^ **)**_**2**_	> 50	> 50
**TT‐(4PyMe** ^**+**^ **I** ^**−**^ **)**_**3**_	> 50	> 50

### 2.6 Docking Studies

To determine the binding mode of the most active compounds within the here‐investigated series, that is, **TT‐(E‐2PyMe** ^**+**^ **I** ^**−**^ **)**, **TT‐(E‐2PyMe** ^**+**^ **I** ^**−**^ **)**_**2**_, **TT‐(E‐2PyMe** ^**+**^ **I** ^**−**^ **)**_**3**_, and **TT‐(E‐4PyMe** ^**+**^ **I** ^**−**^ **)**_**3**_, to the investigated G‐quadruplex models and the control duplex, molecular docking studies were carried out to build structural models for the 1:1 DNA/ligand complexes. As G‐quadruplex and duplex models, PDB‐deposited structures containing either accessible outer G‐quartets and grooves, for the G‐quadruplex models, or accessible intercalative binding sites and grooves, for the duplex model, were selected, that is, those corresponding to hybrid‐2 tel26 G‐quadruplex (PDB 5MVB), parallel pu22 G‐quadruplex (PDB 2L7V), and B‐like duplex ds12 (PDB prepared from 1NAJ). These models allowed us to evaluate the preference of the ligands to recognize the outer G‐quartets or grooves, in the case of G‐quadruplex structures, or intercalative binding sites or grooves, in the case of duplex structures.

**Figure 3:**
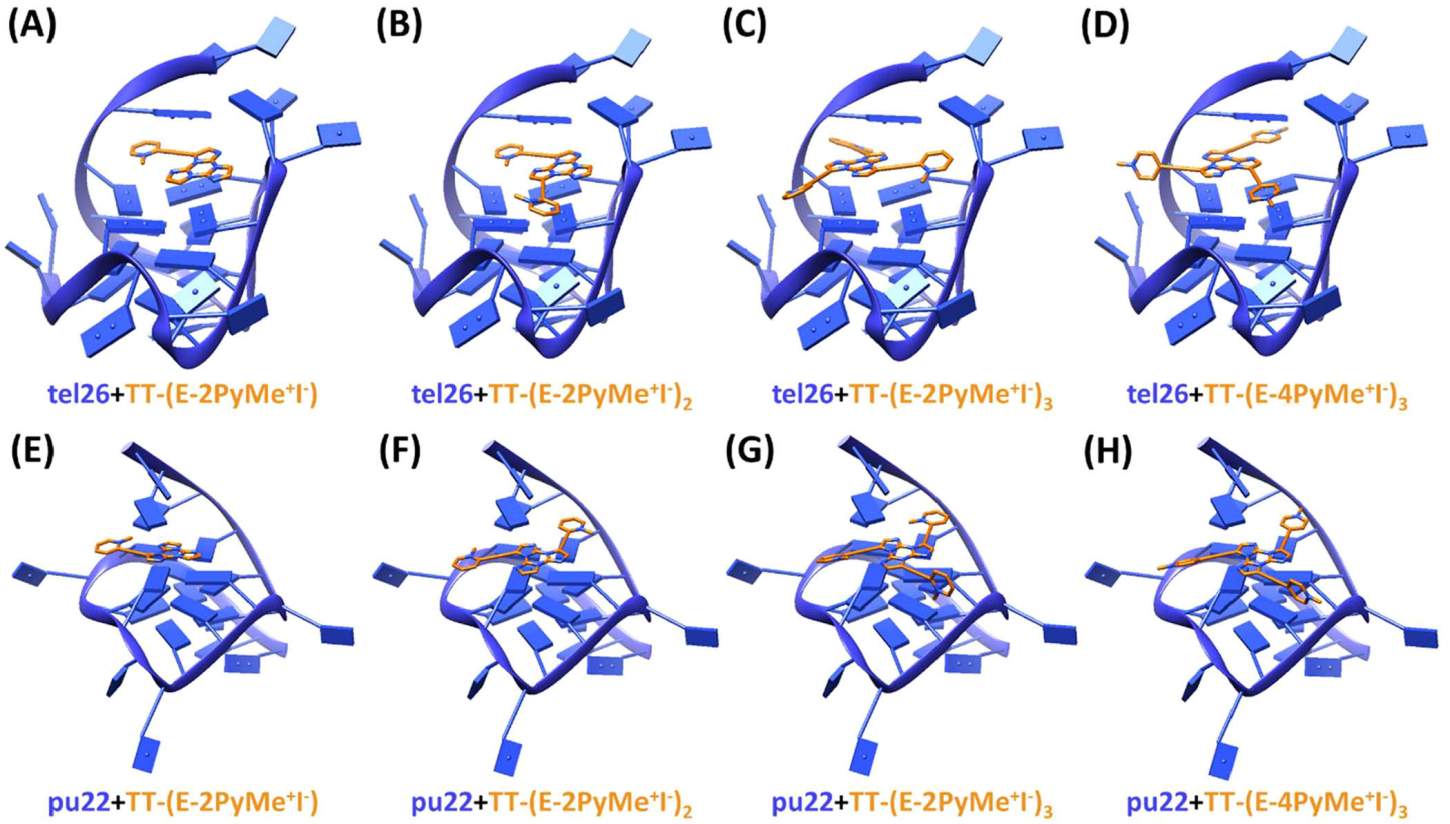
Binding modes (side view) of **TT‐(E‐2PyMe** ^**+**^ **I** ^**–**^ **)**, **TT‐(E‐2PyMe** ^**+**^ **I** ^**–**^ **)**_**2**_, **TT‐(E‐2PyMe** ^**+**^ **I** ^**–**^ **)**_**3**_, and **TT‐(E‐4PyMe** ^**+**^ **I** ^**–**^ **)**_**3**_ when docked to the tel26 G‐quadruplex PDB 5MVB (A, B, C, and D, respectively) and pu22 G‐quadruplex PDB 2L7V (E, F, G, and H, respectively). Ligands and G‐quadruplexes are represented as orange sticks and blue ribbons, respectively. 5′‐ and 3′‐end of the G‐quadruplexes are at the top and bottom, respectively.

**Figure 4:**
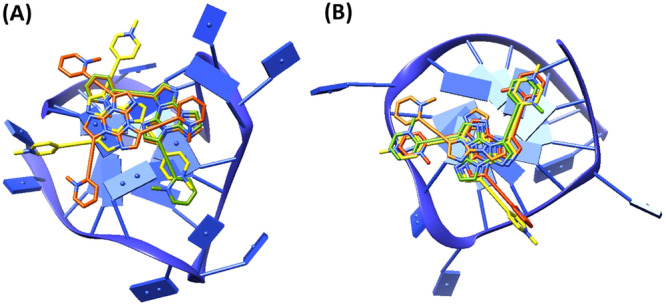
Binding modes (top view) of **TT‐(E‐2PyMe** ^**+**^ **I** ^**–**^ **)**, **TT‐(E‐2PyMe** ^**+**^ **I** ^**–**^ **)**_**2**_, **TT‐(E‐2PyMe** ^**+**^ **I** ^**–**^ **)**_**3**_, and **TT‐(E‐4PyMe** ^**+**^ **I** ^**–**^ **)**_**3**_ when docked to A) tel26 G‐quadruplex PDB 5MVB and B) pu22 G‐quadruplex PDB 2L7V. **TT‐(E‐2PyMe** ^**+**^ **I** ^**–**^ **)**, **TT‐(E‐2PyMe** ^**+**^ **I** ^**–**^ **)**_**2**_, **TT‐(E‐2PyMe** ^**+**^ **I** ^**–**^ **)**_**3**_, and **TT‐(E‐4PyMe** ^**+**^ **I** ^**–**^ **)**_**3**_ are represented as orange, green, red, and yellow sticks, respectively, while the G‐quadruplexes are represented as blue ribbons. For ease of illustration, the first three residues at the 5’‐end of both G‐quadruplexes have been deleted.

All ligands preferentially targeted the outer G‐quartet of the G‐quadruplex models tel26 and pu22 (Figure 3A–H). In detail, the cores of all four ligands were stacked on top of the outer G‐quartet, while the positively charged groups of each ligand pointed to the top edge of the G‐quadruplex grooves, stabilized by electrostatic interactions with the phosphate backbone. Particularly, **TT‐(E‐2PyMe** ^**+**^ **I** ^**–**^ **)** and **TT‐(E‐2PyMe** ^**+**^ **I** ^**–**^ **)**_**2**_ were stacked on G12 and G16 of tel26 G‐quadruplex, and **TT‐(E‐2PyMe** ^**+**^ **I** ^**–**^ **)**_**3**_ and **TT‐(E‐4PyMe** ^**+**^ **I** ^**–**^ **)**_**3**_ were stacked on G4 of tel26 (Figure 4A), while more similar binding poses were found for all four compounds on pu22 G‐quadruplex which were all stacked on G11 and G16 (Figure 4B).

In the case of the duplex model ds12, the monosubstituted compound **TT‐(E‐2PyMe** ^**+**^ **I** ^**–**^ **)** was able to intercalate in the duplex (Supporting Information S2: Figure S17A), but **TT‐(E‐2PyMe** ^**+**^ **I** ^**–**^ **)**_**2**_ and the trisubstituted compounds, **TT‐(E‐2PyMe** ^**+**^ **I** ^**–**^ **)**_**3**_ and **TT‐(E‐4PyMe** ^**+**^ **I** ^**–**^ **)**_**3**_, even in the presence of the intercalative binding site, were unable to target this site, in turn interacting with the minor groove of the duplex (Supporting Information S2: Figure S17B–D), probably as a consequence of the steric hindrance of the pendant groups.

Notably, by analysis of the binding energies obtained for the found binding poses (Supporting Information S2: Table S1), it emerged the following trend of affinity for both tel26 and pu22 G‐quadruplexes: **TT‐(E‐2PyMe** ^**+**^ **I** ^**–**^ **)**_**3**_ > **TT‐(E‐4PyMe** ^**+**^ **I** ^**–**^ **)**_**3**_ > **TT‐(E‐2PyMe** ^**+**^ **I** ^**–**^ **)**_**2**_ > **TT‐(E‐2PyMe** ^**+**^ **I** ^**–**^ **)**, which perfectly matches the affinity trend observed in the G4‐CPG assays, as well as the stabilizing ability trend found in CD experiments. On the other hand, in the case of the ds12 duplex, the affinity trend was as follows: **TT‐(E‐4PyMe** ^**+**^ **I** ^**–**^ **)**_**3**_ > **TT‐(E‐2PyMe** ^**+**^ **I** ^**–**^ **)**_**3**_ > **TT‐(E‐2PyMe** ^**+**^ **I** ^**–**^ **)**_**2**_ > **TT‐(E‐2PyMe** ^**+**^ **I** ^**–**^ **)**, which, in agreement with the above biophysical data, proved the lower affinity for the duplex control of the mono‐ and disubstituted compounds compared to the trisubstituted compounds.

## 3 Conclusions

In this study, new **TT** derivatives functionalized with one, two, or three methylated ethynylpyridine moieties have been prepared and studied as G‐quadruplex stabilizers. The insertion of the triple bond between the tricyclic system and the methylated pyridines was intended not only to impose a longer distance between the positive charge and the aromatic core but also to extend the π‐electron system responsible for π–π interactions with the G‐quartets, thus enabling better G‐quadruplex stabilization.

The compounds have been tested for their ability to bind G‐quadruplex structures from both telomeric and oncogene promoter sequences by chromatographic, spectroscopic, and electrophoretic techniques as well as by molecular docking. The obtained results demonstrated the ability of the investigated compounds to specifically interact with G‐quadruplexes, particularly targeting the outer G‐quartets by their cores and the top edge of G‐quadruplex grooves by the positively charged pyridinium groups.

Generally, the trisubstituted compounds showed higher stabilizing properties but lower G‐quadruplex selectivity than the related di‐ and mono‐substituted ones. Interestingly, the di‐ and tri‐substituted derivatives were able to induce a conformational conversion of the telomeric G‐quadruplex model from hybrid‐2 to hybrid‐1 topology at 20°C, and, even more notably, the trisubstituted ones were found able to induce a conformational change on telomeric G‐quadruplex from hybrid‐1 to a parallel topology at 70°C, which remained stable even at 95°C, emerging as potential tools for converting the G‐quadruplex fold of telomeric sequences to a specific topology.

Among the studied compounds, **TT‐(E‐2PyMe** ^**+**^ **I** ^**–**^ **)**, **TT‐(E‐2PyMe** ^**+**^ **I** ^**–**^ **)**_**2**_, **TT‐(E‐2PyMe** ^**+**^ **I** ^**–**^ **)**_**3**_, and **TT‐(E‐4PyMe** ^**+**^ **I** ^**–**^ **)**_**3**_ proved to be the most antiproliferative species in the series on the tested cancer cell lines, with IC_50_ values in the micromolar range. Thus, these preliminary biological assays showed a potential correlation between the antiproliferative activity found for some of the compounds and their ability to target and stabilize G‐quadruplexes, especially in comparison with their previously studied analogs. However, more detailed biological assays, particularly aimed at evaluating the ability of the active compounds to specifically recognize nuclear G‐quadruplexes, will be part of future investigations in which not only the here most active compounds will be included but also improved analogs thereof, designed on the basis of the here‐obtained interaction data.

Altogether the biophysical experiments, also in agreement with the biological assays, underlined the importance of the introduction of an alkyne linker between the triimidazole core and the methylpyridinium group, which proved to be beneficial to increase both the stabilizing effects on G‐quadruplexes and the anticancer activity compared with the previously studied analogs lacking the alkyne linker.

## 4 Materials and Methods

### 4.1 Chemical Synthesis

#### 4.1.1 General Methods

All reagents and solvents were purchased from specialized chemical suppliers and used without further purification unless otherwise stated. **TT** [37], **TT‐Br** [11, 21], **TT‐Br**_**2**_ [11, 21], **TT‐Br**_**3**_ [11, 21], **TT‐E‐2Py** [38], and **TT‐E‐4Py** [38] were prepared according to previously reported procedures. All solvents used for HPLC analysis were of LC‐MS grade. Thin layer chromatography (TLC) was performed using Merck silica gel 60 F254 precoated plates. Column chromatography was carried out with Biotage apparatus equipped with silica gel Sfär Silica D Duo columns or by flash chromatography performed using Merck 60 silica gel. ^1^H and ^13^C NMR spectra were recorded at 300 K on a Bruker AVANCE‐400 instrument (400 MHz) equipped with a BBI 1H/Multinuclear Probe. Chemical shifts are reported in parts per million (ppm) and are referenced to the residual solvent peak (DMSO, ^1^H: *δ* = 2.50 ppm, ^13^C: *δ* = 39.5 ppm; D_2_O ^1^H: *δ* = 4.80 ppm; CD_2_Cl_2_, ^1^H: *δ* = 5.32 ppm, ^13^C: *δ* = 54.0 ppm). Coupling constants (*J*) are given in hertz (Hz) and are quoted to the nearest 0.5 Hz. Peak multiplicities are described in the following way: s, singlet; d, doublet; t, triplet. Mass spectra were recorded on a Thermo Fisher LCQ Fleet Ion Trap Mass Spectrometer equipped with UltiMate 3000 high‐performance liquid chromatography (HPLC) system. For the HPLC elutions, the following solutions were used: 95/5 H_2_O/CH_3_CN (v/v) + 0.1% formic acid (solution A), 5/95 H_2_O/CH_3_CN (v/v) + 0.1% formic acid (solution B); Acquity UPLC 2.1 × 100 mm column, BEH C18 (1.7 μm; flow 0.6 mL/min) coupled with an Acquity QDa Water mass spectrometer (probe temperature: 600°C; ESI capillary voltage 1.5 V; cone voltage 15 V; mass range 60–1000). HRMS spectra were obtained using Synapt G2‐Si QTof mass spectrometer (Waters) with Zspray ESI‐probe for electrospray ionization (Waters) (Supporting Information S2: Figures S18–S54).

The InChI codes of the investigated compounds, together with some biological activity data, are provided as Supporting Information.

#### 4.1.2 Synthesis of 3,7‐Bis(Pyridin‐2‐ylethynyl)Triimidazo[1,2‐*a*:1′,2′‐*c*:1″,2″‐*e*][1,3,5]Triazine (**TT‐(E‐2Py)**_2_)

**TT‐Br**_**2**_ (200 mg, 0.562 mmol), Pd(PPh_3_)_2_Cl_2_ (20 mg, 0.028 mmol), dry *N*,*N*‐diisopropylethylamine (DIPEA, 0.8 mL), and dry *N,N*‐dimethylformamide (DMF, 10 mL) were introduced into a 50 mL dry Schlenk flask equipped with a magnetic stir bar under Ar atmosphere. To this mixture, 2‐ethynylpyridine (0.227 mL, 2.26 mmol) was further added, and the system was heated at 90°C and kept in a static Ar atmosphere for 16 h under stirring. The resulting brown reaction mixture was then cooled to room temperature and evaporated to dryness. The crude reaction mixture was purified with Biotage apparatus equipped with silica gel Sfär Silica D Duo using a dichloromethane (DCM)/CH_3_CN mixture as eluent (*R*_f_ = 0.5 on TLC in DCM/CH_3_CN 8:2, v/v). Further precipitation from CH_3_CN gave the pure **TT‐(E‐2Py)**_**2**_ product as a white solid (0.060 g, 0.150 mmol, yield: 27%). ^1^H NMR (400 MHz, CD_2_Cl_2_, 298 K): *δ* 8.64 (d, *J* = 4.80 Hz, 2H), 7.86 (d, *J* = 1.69 Hz, 1H), 7.77 (dt, *J* = 1.57 Hz, 2H), 7.71 (m, 2H), 7.61 (s, 2H), 7.41 (d, *J* = 1.69 Hz, 1H), 7.32 (m, *2*H). ^13^C NMR (100 MHz, CD_2_Cl_2_, 298 K): δ 150.9 (2CH), 143.2 (C), 143.1 (CH), 136.8 (2CH), 136.4 (C), 136.3 (CH), 136.2 (CH), 136.0 (C), 135.7 (C), 130.4 (CH), 128.0 (2CH), 124.0 (2CH), 112.2 (CH), 110.6 (C), 110.3 (C), 97.5 (C), 97.4 (C), 76.3 (C), 76.1 (C). ESI‐MS: *m/z* 401.31 [M+H]^+^.

#### 4.1.3 Synthesis of 3,7,11‐Tris(Pyridin‐2‐ylethynyl)Triimidazo[1,2‐*a*:1′,2′‐*c*:1″,2″‐*e*][1,3,5]Triazine (**TT‐(E‐2Py)**_3_)

**TT‐Br**_**3**_ (200 mg, 0.46 mmol), Pd(PPh_3_)_2_Cl_2_ (97 mg, 0.14 mmol), dry DIPEA (0.7 mL), and dry DMF (10 mL) were introduced into a 50 mL dry Schlenk flask equipped with a magnetic stir bar under Ar atmosphere. To this mixture, 2‐ethynylpyridine (0.28 mL, 2.8 mmol) was added, and the system was heated at 90°C and kept in a static Ar atmosphere for 16 h under stirring. The resulting brown reaction mixture was then cooled to room temperature and evaporated to dryness. The crude reaction mixture was purified by gravimetric chromatography on SiO_2_ using a mixture of DCM/CH_3_CN as eluent (TLC *R*_f_ = 0.5 in DCM/CH_3_CN 9:1, v/v). Further precipitation from DCM/*n*‐hexane gave the pure **TT‐(E‐2Py)**_**3**_ product as a white solid (0.065 g, 0.13 mmol, yield: 28%). ^1^H NMR (400 MHz, CD_2_Cl_2_, 298 K): *δ* 8.66 (d, *J* = 4.80 Hz, 3H), 7.78 (dt, *J* = 7.70 and 1.60 Hz, 3H), 7.73 (s, 3H), 7.71 (d, *J* = 4.80 Hz, 3H), 7.33 (m, 3H). ^13^C NMR (100 MHz, CD_2_Cl_2_, 298 K): *δ* 150.9 (CH), 143.2 (C), 136.8 (CH), 136.6 (CH), 135.6 (C), 128.0 (CH), 124.1 (CH), 110.5 (C), 97.8 (C), 76.0 (C). ESI‐MS: *m/z* 502.43 [M+H]^+^.

#### 4.1.4 Synthesis of 3,7‐Bis(Pyridin‐4‐ylethynyl)Triimidazo[1,2‐*a*:1′,2′‐*c*:1″,2″‐*e*][1,3,5]Triazine (**TT‐(E‐4Py)**_2_)

**TT‐Br**_**2**_ (200 mg, 0.562 mmol), Pd(PPh_3_)_2_Cl_2_ (20 mg, 0.028 mmol), dry DIPEA (0.8 mL), and dry DMF (10 mL) were introduced into a 50 mL dry Schlenk flask equipped with a magnetic stir bar under Ar atmosphere. To this mixture, 4‐ethynylpyridine (0.228 mL, 2.26 mmol) was then added, and the system was heated at 90°C and kept in a static Ar atmosphere for 16 h under stirring. The resulting brown reaction mixture was then cooled to room temperature and evaporated to dryness. The crude reaction mixture was purified with Biotage apparatus equipped with silica gel Sfär Silica D Duo with a gradient of isopropyl alcohol (IPA) in DCM as eluent (TLC *R*_f_ = 0.5 in DCM/IPA 9:1, v/v). Further precipitation from CH_3_CN gave the pure **TT‐(E‐4Py)**_**2**_ product as a white solid (0.080 g, 0.199 mmol, yield: 35%). ^1^H NMR (400 MHz, DMSO‐d6, 298 K): *δ* 8.69 (m, 4H), 8.09 (d, *J* = 1.69 Hz, 1H), 7.89 (s, 1H), 7.84 (s, 1H), 7.59 (m, *4*H), 7.43 (d, *J* = 1.69 Hz, 1H). ^13^C NMR (100 MHz, CD_2_Cl_2_, 298 K): δ 150.6 (CH), 136.1 (CH), 136.0 (CH), 135.7 (C), 135.6 (C), 135.7 (C), 130.8 (C), 130.7 (C), 130.4 (CH), 125.4 (CH), 125.3 (CH), 112.1 (CH), 110.5 (C), 110.2 (C), 95.7 (C), 95.6 (C), 81.1 (C), 80.9 (C). ESI‐MS: *m/z* 401.22 [M+H]^+^.

#### 4.1.5 Synthesis of 3,7,11‐Tris(Pyridin‐4‐ylethynyl)Triimidazo[1,2‐*a*:1′,2′‐*c*:1″,2″‐*e*][1,3,5]Triazine (**TT‐(E‐4Py)**_3_)

**TT‐Br**_**3**_ (200 mg, 0.46 mmol), Pd(PPh_3_)_2_Cl_2_ (97 mg, 0.14 mmol), dry DIPEA (0.7 mL), and dry DMF (10 mL) were introduced into a 50 mL dry Schlenk flask equipped with a magnetic stir bar under Ar atmosphere. To this mixture, 4‐ethynylpyridine (0.280 mL, 2.76 mmol) was added, and the system was then heated at 90°C and kept in a static Ar atmosphere for 16 h under stirring. The resulting brown reaction mixture was then cooled to room temperature and evaporated to dryness. The crude reaction mixture was purified with Biotage apparatus equipped with silica gel Sfär Silica D Duo using a gradient of IPA in DCM as eluent (TLC *R*_f_ = 0.5 in DCM/IPA 8:2, v/v). Further precipitation from CH_3_CN gave the pure **TT‐(E‐4Py)**_**3**_ product as a white solid (0.050 g, 0.099 mmol, yield: 22%). ^1^H NMR (400 MHz, CD_2_Cl_2_, 298 K): *δ* 8.65 (m, 6H), 7.69 (s, 3H), 7.52 (m, 6H). ^13^C NMR (100 MHz, CD_2_Cl_2_, 298 K): δ 150.6 (CH), 136.3 (CH), 135.8 (C), 130.7 (C), 125.4 (CH), 110.4 (C), 96.0 (C), 80.8 (C). ESI‐MS: *m/z* 502.06 [M+H]^+^.

#### 4.1.6 Synthesis of 1‐Methyl‐2‐(Triimidazo[1,2‐*a*:1′,2′‐*c*:1″,2″‐*e*][1,3,5]Triazin‐3‐ylethynyl)Pyridin‐1‐ium Iodide (**TT‐E‐2PyMe** ^+^ **I** ^‐^)

**TT‐E‐2PyMe** ^**+**^ **I** ^**‒**^ was prepared by methylation of **TT‐E‐2Py**. In a typical reaction, **TT‐E‐2Py** (100 mg, 0.334 mmol) and CH_3_I (7 mL) were transferred into a 20 mL closed vial equipped with a magnetic stir bar. The system was stirred at 60°C for 3 h. The reaction mixture was then filtered on a Büchner funnel, and the obtained precipitate was exhaustively washed with DCM to give the pure **TT‐E‐2PyMe** ^**+**^ **I** ^**‒**^ as a yellow solid (127 mg, 0.288 mmol, 86% yield). ^1^H NMR (400 MHz, D_2_O, 298 K): *δ* 8.85 (d, *J* = 6.19 Hz, 1H), 8.51 (t, *J* = 7.56 Hz, 1H), 8.29 (d, *J* = 7.16 Hz, 1H), 7.97 (t, *J* = 7.20 Hz, 1H), 7.93 (s, 1H), 7.92 (d, *J* = 1.78 Hz, 1H), 7.91 (d, *J* = 1.84 Hz, 1H), 7.36 (d, *J* = 1.78 Hz, 1H), 7.33 (d, *J* = 1.84 Hz, 1H), 4.54 (s, 3H). ^13^C NMR (100 MHz, D_2_O, 298 K): δ 146.6 (CH), 144.7 (CH), 138.6 (CH), 137.3 (C), 137.1 (C), 136.0 (C), 135.0 (C), 131.3 (CH), 129.0 (CH), 128.8 (CH), 126.9 (CH), 112.2 (CH), 112.2 (CH), 107.1 (C), 92.5 (C), 88.1 (C), 47.5 (CH_3_). HRMS (ESI‐positive ion mode): calcd. for C_17_H_12_N_7_^+^ 314.1149, found *m/z* 314.1153 ([M]^+^).

#### 4.1.7 Synthesis of 2,2′‐(Triimidazo[1,2‐*a*:1′,2′‐*c*:1″,2″‐*e*][1,3,5]Triazine‐3,7‐diylbis(Ethyne‐2,1‐diyl))Bis(1‐Methylpyridin‐1‐ium) Iodide (**TT‐(E‐2PyMe** ^+^ **I** ^‐^ **)**_2_)

**TT‐(E‐2PyMe** ^**+**^ **I** ^**‒**^ **)**_**2**_ was prepared by methylation of **TT‐(E‐2Py)**_**2**_. In a typical reaction, **TT‐(E‐2Py)**_**2**_ (100 mg, 0.25 mmol) and CH_3_I (4 mL) were transferred into a 20 mL closed vial equipped with a magnetic stir bar. The system was stirred at 60°C for 7 h. The reaction mixture was then filtered on a Büchner funnel, and the obtained precipitate was washed with DCM to give pure **TT‐(E‐2PyMe** ^**+**^ **I** ^**‒**^ **)**_**2**_ as a yellow solid (160 mg, 0.234 mmol, 94% yield). ^1^H NMR (400 MHz, DMSO‐d6, 298 K): *δ* 9.16 (d, *J* = 6.09 Hz, 2H), 8.63 (t, *J* = 7.84 Hz, 2H), 8.38 (d, *J* = 7.88 Hz, 2H), 8.25 (m, 3H), 8.13 (t, *J* = 6.70 Hz, 2H), 7.53 (d, *J* = 1.78 Hz, 1H), 4.63 (s, 3H), 4.60 (s, 3H). ^13^C NMR (100 MHz, DMSO‐d6, 298 K): *δ* 147.2 (CH), 144.7 (CH), 139.8 (C), 139.3 (C), 138.0 (C), 137.0 (C), 136.7 (CH), 135.4 (C), 130.6 (2 CH), 129.5 (CH), 126.7 (CH), 112.3 (CH), 106.3 (2 C), 92.8 (2 C), 88.9 (2C), 47.6 (CH_3_), 47.5 (CH_3_). HRMS (ESI‐positive ion mode): calcd. for C_25_H_18_N_8_^2+^ 430.1643, found *m/z* 215.0823 ([M]^2+^).

#### 4.1.8 Synthesis of 2,2′,2″‐(Triimidazo[1,2‐*a*:1′,2′‐*c*:1″,2″‐*e*][1,3,5]Triazine‐3,7,11‐triyltris(Ethyne‐2,1‐diyl))Tris(1‐Methylpyridin‐1‐ium) Iodide (**TT‐(E‐2PyMe** ^+^ **I** ^‐^ **)**_3_)

**TT‐(E‐2PyMe** ^**+**^ **I** ^**‒**^ **)**_**3**_ was prepared by methylation of **TT‐(E‐2Py)**_**3**_. In a typical reaction, **TT‐(E‐2Py)**_**3**_ (130 mg, 0.26 mmol) and CH_3_I (4 mL) were transferred into a 20 mL closed vial equipped with a magnetic stir bar. The system was stirred at 60°C for 7 h. The reaction mixture was then filtered on a Büchner funnel, and the obtained precipitate was washed with DCM to give pure **TT‐(E‐2PyMe** ^**+**^ **I** ^**‒**^ **)**_**3**_ as a yellow solid (220 mg, 90% yield). ^1^H NMR (400 MHz, DMSO‐d6, 298 K): *δ* 9.19 (d, *J* = 6.06 Hz, 3H), 8.65 (t, *J* = 7.82 Hz, 3H), 8.41 (d, *J* = 7.96 Hz, 3H), 8.37 (s, 3H), 8.15 (t, *J* = 8.16 Hz, 3H), 4.61 (s, 9H). ^13^C NMR (100 MHz, DMSO‐d6, 298 K): *δ* 147.6 (CH), 144.8 (CH), 139.8 (C), 137.2 (C), 136.5 (CH), 130.8 (CH), 127.0 (C), 106.7 (CH), 91.8 (C), 89.2 (C), 47.6 (CH_3_). HRMS (ESI‐positive ion mode): calcd. for C_33_H_24_N_9_^3+^ 546.2138, found *m/z* 182.0717 ([M]^3+^).

#### 4.1.9 Synthesis of 1‐Methyl‐4‐(Triimidazo[1,2‐a:1′,2′‐c:1″,2″‐e][1,3,5]Triazin‐3‐ylethynyl)Pyridin‐1‐ium Iodide (**TT‐E‐4PyMe** ^+^ **I** ^‐^)

**TT‐E‐4PyMe** ^**+**^ **I** ^**‒**^ was prepared by methylation of **TT‐E‐4Py**. In a typical reaction, **TT‐E‐4Py** (100 mg, 0.334 mmol) and CH_3_I (7 mL) were transferred into a 20 mL closed vial equipped with a magnetic stir bar. The system was stirred at 60°C for 3 h. The reaction mixture was then filtered on a Büchner funnel, and the obtained precipitate was exhaustively washed with DCM to give pure **TT‐E‐4PyMe** ^**+**^ **I** ^**‒**^ as a yellow solid (130 mg, 0.295 mmol, 88% yield). ^1^H NMR (400 MHz, D_2_O, 298 K): *δ* 8.74 (d, *J* = 6.70 Hz, 2H), 8.11 (d, *J* = 6.70 Hz, 2H), 7.88 (d, *J* = 1.71 Hz, 1H), 7.85 (d, *J* = 1.76 Hz, 1H), 7.80 (s, 1H), 7.34 (d, *J* = 1.71 Hz, 1H), 7.29 (d, *J* = 1.76 Hz, 1H), 4.34 (s, 3H). ^13^C NMR (100 MHz, D_2_O, 298 K): *δ* 144.9 (CH), 138.7 (C), 138.4 (CH), 136.8 (C), 135.8 (C), 135.0 (C), 128.9 (CH), 128.9 (CH), 128.6 (CH), 112.3 (CH), 112.2 (CH), 107.9 (C), 93.7 (C), 89.2 (C), 48.0 (CH_3_). HRMS (ESI‐positive ion mode): calcd. for C_17_H_12_N_7_^+^ 314.1149, found *m/z* 314.1150 ([M]^+^).

#### 4.1.10 Synthesis of 4,4′‐(Triimidazo[1,2‐*a*:1′,2′‐*c*:1″,2″‐*e*][1,3,5]Triazine‐3,7‐diylbis(Ethyne‐2,1‐diyl))Bis(1‐Methylpyridin‐1‐ium) Iodide (**TT‐(E‐4PyMe** ^+^ **I** ^‐^ **)**_2_)

**TT‐(E‐4PyMe** ^**+**^ **I** ^**‒**^ **)**_**2**_ was prepared by methylation of **TT‐(E‐4Py)**_**2**_. In a typical reaction, **TT‐(E‐4Py)**_**2**_. (100 mg, 0.249 mmol) and CH_3_I (7 mL) were transferred into a 20 mL closed vial equipped with a magnetic stir bar. The system was stirred at 60°C for 3 h. The reaction mixture was then filtered on a Büchner funnel, and the obtained precipitate was exhaustively washed with DCM to give pure **TT‐(E‐4PyMe** ^**+**^ **I** ^**‒**^ **)**_**2**_ as a yellow solid (140 mg, 0.205 mmol, 82% yield). ^1^H NMR (400 MHz, D_2_O, 298 K): *δ* 8.77 (m, 4H), 8.18 (m, 4H), 8.02 (d, *J* = 1.62 Hz, 1H), 7.96 (s, 1H), 7.91 (s, 1H), 7.45 (d, *J* = 1.62 Hz, 1H), 4.37 (s, 6H). ^13^C NMR (100 MHz, D_2_O, 298 K): *δ* 145.7 (CH), 139.5 (CH), 139.4 (CH), 139.2 (CH), 137.8 (C), 137.1 (C), 136.2 (C), 130.1 (C), 129.5 (CH), 129.4 (CH), 113.3 (CH), 109.1 (C), 94.6 (C), 89.7 (C), 48.7 (CH_3_). HRMS (ESI‐positive ion mode): calcd. for C_25_H_18_N_8_^2+^ 430.1643, found *m/z* 215.0819 ([M]^2+^).

#### 4.1.11 Synthesis of 4,4′,4″‐(Triimidazo[1,2‐*a*:1′,2′‐*c*:1″,2″‐*e*][1,3,5]Triazine‐3,7,11‐triyltris(Ethyne‐2,1‐diyl))Tris(1‐Methylpyridin‐1‐ium) Iodide (**TT‐(E‐4PyMe** ^+^ **I** ^‐^ **)**_3_)

**TT‐(E‐4PyMe** ^**+**^ **I** ^**‒**^ **)**_**3**_ was prepared by methylation of **TT‐(E‐4Py)**_**3**_. In a typical reaction, **TT‐(E‐4Py)**_**3**_ (100 mg, 0.2 mmol) and CH_3_I (7 mL) were transferred into a 20 mL closed vial equipped with a magnetic stir bar. The system was stirred at 60°C for 3 h. The reaction mixture was then filtered on a Büchner, and the obtained precipitate was exhaustively washed with DCM to give pure **TT‐(E‐4PyMe** ^**+**^ **I** ^**‒**^ **)**_**3**_ as a yellow solid (150 mg, 0.162 mmol, 81% yield). ^1^H NMR (400 MHz, DMSO‐d6, 298 K): *δ* 9.04 (d, *J* = 6.38 Hz, 6H), 8.26 (d, *J* = 6.38 Hz, 6H), 8.20 (s, 3H), 4.37 (s, 9H). ^13^C NMR (100 MHz, DMSO‐d6, 298 K): *δ* 145.8 (CH), 138.7 (C), 137.2 (CH), 128.0 (CH), 107.4 (C), 93.9 (C), 89.4 (C), 47.9 (CH_3_). HRMS (ESI‐positive ion mode): calcd. for C_33_H_24_N_9_^3+^, 546.2138, found *m/z* 182.0712 ([M]^3+^).

### 4.2 G4‐CPG Assay

Stock solutions (4 mM) of each compound were prepared by dissolving a known amount of the tested ligand in pure DMSO. A defined volume was taken from the initial stock solution to obtain a 60 μM compound solution in 50 mM KCl, 10% DMSO, and 10% CH_3_CH_2_OH aq. solution. The detailed general procedure adopted for the assays is as follows: weighed amounts of the nude CPG, G‐quadruplex‐functionalized and duplex‐functionalized CPG supports (ca. 8 mg) [20] were left separately in contact with 300 μL of the compound solution in a polypropylene column (4 mL volume, Alltech) equipped with a polytetrafluoroethylene frit (10 μm porosity), a stopcock, and a cap. After incubation on a vibrating shaker for 4 min, each support was washed with defined volumes of the washing solution (50 mM KCl, 10% DMSO, 10% CH_3_CH_2_OH aq. solution) or the releasing solution (2.5 M CaCl_2_, 15% DMSO aq. solution or pure DMSO) and all the eluted fractions were separately analyzed by UV measurements. After treatment with the releasing solution, inducing G‐quadruplexes and duplex full denaturation, the DNA‐functionalized supports were resuspended in the washing solution and then subjected to annealing procedure—by taking the suspensions at 75°C for 5 min and then slowly cooling them to r.t.—to allow the correct DNA folding for the subsequent reuse of the supports.

The UV measurements were performed on a JASCO V‐550 UV‐vis spectrophotometer. A quartz cuvette with a path length of 1 cm was used. The UV quantification of the compounds was determined by measuring the absorbance relative to the *λ*_max_ characteristic of each compound and referring it to the corresponding calibration curves. The errors associated with the % of bound ligand are within ± 2%.

### 4.3 CD Analysis

CD spectra were recorded in a quartz cuvette with a path length of 1 cm, on a Jasco J‐715 spectropolarimeter equipped with a Peltier‐type temperature control system (model PTC‐348WI). The spectra were recorded at 20°C in the range 220–600 nm, 200 nm/min scanning speed, and 2.0 nm bandwidth and were corrected by subtraction of the background scan with buffer. All the spectra were averaged over three scans. The oligonucleotides d[(TTAGGG)_4_TT] (tel26), d(TGAGGGTGGGTAGGGTGGGTAA) (pu22), and d(CGCGAATTCGCG) (ds12) were purchased from Biomers as HPLC‐purified, desalted, and lyophilized compounds with a purity > 99%. The oligonucleotides tel26 and ds12 were dissolved in a 20 mM KCl, 5 mM potassium phosphate buffer (pH 7), while pu22 was dissolved in a 10 mM Tris–HCl buffer (pH 7), to obtain 2 μM solutions, which were then annealed by heating at 95°C for 5 min, followed by slow cooling to r.t. CD titrations were obtained by adding increasing amounts of each compound (up to 2 molar equivalents, corresponding to a 4 μM compound solution, or up to 10 molar equivalents, corresponding to a 20 μM compound for **TT‐(E‐2PyMe** ^**+**^ **I** ^**‐**^ **)**_**3**_ to the oligonucleotide solutions. For the CD melting experiments, the ellipticity was recorded at 290 nm for tel26, 263 nm for pu22, and 253 nm for ds12 with a temperature scan rate of 1°C/min, in the range 10°C–95°C. Particularly, in CD experiments, pu22 was analyzed in a metal cation‐free buffer, considering that even in the presence of very low amounts of metal cations, the pu22 G‐quadruplex is so stable that its *T*_m_ value, as well as the related Δ*T*_m_ values in the presence of each ligand, cannot be accurately determined.

### 4.4 Gel Electrophoresis Experiments

The 1:2 DNA/ligand mixtures and free oligonucleotide samples were loaded and resolved on native 20% polyacrylamide (19:1 acrylamide/bisacrylamide) gel. Tris‐Borate‐EDTA (TBE 1x), supplemented with 25 mM KCl for tel26 and ds12, was used as running buffer. The oligonucleotide samples were loaded on gels at 2 µM (or 1 µM for tel46) concentration in 20 mM KCl, 5 mM potassium phosphate buffer (pH 7) for tel26, ds12, and tel46 or 10 mM Tris–HCl buffer (pH 7) for pu22. No migration marker was used. Samples were electrophoresed for 2.5 h at 100 V at r.t. The bands were visualized by a UV transilluminator (BioRad ChemiDoc XRS) using GelGreen staining (30 min incubation). The oligonucleotide d[AGGG(TTAGGG)_7_] (tel46) was purchased from Biomers as HPLC‐purified, desalted, and lyophilized compound with a purity of > 99%.

### 4.5 Biological Assays

#### 4.5.1 Cell Cultures and Cytotoxicity Assays

Human HeLa CCL‐2 adenocarcinoma cells and human MCF7 breast cancer cells were obtained from the American Type Culture Collection (ATCC, Manassas, VA, USA) and cultured in high‐glucose Dulbecco's modified Eagle's medium (DMEM) supplemented with 10% fetal bovine serum (FBS), 1% antibiotics (pen/strep), and 1% l‐glutamine at 37°C in a humidified atmosphere containing 5% CO_2_ as previously described [39]. To perform MTT assays, cells were seeded into 96‐well plates at a density of 5 × 10^3^ cells/well. After 24 h, the cell supernatant was replaced with fresh medium containing increasing concentrations (up to 50 µM) of the tested compounds. Subsequently, cells were cultured for different time intervals (24, 48, and 72 h). After incubation, MTT assays were performed as previously described [40]. Briefly, cell culture supernatants were removed, and cells were incubated with 0.5 mg/mL MTT reagent dissolved in DMEM medium without red phenol (100 μL/well). After 4 h of incubation at 37°C, the resulting insoluble formazan salts were solubilized in anhydrous isopropanol containing 0.01 M HCl and quantified by measuring the absorbance at 570 nm on an automated plate reader spectrophotometer (Synergy H4 Hybrid Microplate Reader, BioTek Instruments Inc., Winooski, VT). Cell viability was expressed as the mean of the percentage values obtained by comparison with control untreated cells.

#### 4.5.2 Statistical Analyses

Statistical analyses were performed by using a Student's *t*‐test. Significant differences were indicated as *p ≤ 0.05.

### 4.6 Molecular Docking

The G‐quadruplex‐forming oligonucleotides tel26 and pu22 were prepared using as starting models the NMR‐deposited structure of the complex tel26/Auoxo6 (PDB 5MVB) [41] and pu22/Quindoline (PDB 2L7V) [42], respectively, from which the bound ligands were removed. The bimolecular duplex‐forming oligonucleotide ds12 was prepared starting from the NMR deposited structure of ds12 (PDB 1NAJ) in which an intercalative binding site between C1:G24 and G2:C23 base pairs was created, using the crystallographic bimolecular duplex structure complexed with the intercalator ligand ellipticine as a template (PDB 1Z3F) [43].

Molecular docking calculations were carried out using AutoDock Vina with the aid of its graphical user interface AutoDockTools [44, 45]. As a control of the docking method, the redocking of Quindoline in the PDB 2L7V was performed before carrying out docking calculations with the here‐investigated compounds, showing no relevant differences between the pose of the ligand in the NMR and docking structures (Supporting Information S2: Figure S55). The ligands and DNA targets were prepared by use of AutoDockTools and UCSF Chimera by assigning bond orders, adding hydrogen atoms, and generating the appropriate protonation states. The ligands and targets were then converted to proper Autodock PDBQT file formats and the Gasteiger charges were assigned. The 3D grid box dimensions were defined, including the whole DNA macromolecules. The docking area was centered on the DNA center of mass and grid boxes of 110 Å × 100 Å × 80 Å, 100 Å × 90 Å × 60 Å, and 60 Å × 120 Å × 60 Å for tel26, pu22, and ds12, respectively, with a 0.375 Å spacing. Twenty docking poses were generated by using as docking parameters: seed = random, exhaustiveness = 24 for each DNA/ligand system. Docking poses were clustered on the basis of their root‐mean‐square deviation and ranked on the basis of binding energy. Molecular modeling figures were drawn by UCSF Chimera.

## Supporting information

ArchPharm SupplMat InChI.

Supp Inf cyclic triimidazoles final revised.

## Data Availability

The data that support the findings of this study are available on request from the corresponding author. The data are not publicly available due to privacy or ethical restrictions.
