# Supplementary material for: Combining Cyclic Triimidazo Triazine Core With Ethynyl‐N‐Methyl‐Pyridinium Groups for Targeting G‐Quadruplex Structures
Source: Arch Pharm (Weinheim). 2025 Jul 2;358(7):e70037. doi: 10.1002/ardp.70037 (PMC12223358; doi:10.1002/ardp.70037)
Supplement: Supplementary file 1 — ArchPharm SupplMat InChI. [file ARDP-358-e70037-s001.pdf]

ARCH PHARM
Archiv der Pharmazie

Supplemental Material: Novel Compounds and Biological Screening Results

(PLEASE REPLACE ALL INFORMATION AND ADVICE GIVEN IN BLUE BEFORE SUBMISSION)!

Title of Manuscript

Combining cyclic triimidazo triazine core with ethynyl-*N*-methyl-pyridinium groups for targeting G-quadruplex structures

Authors

Chiara Platella,[a,§] Stefano Di Ciolo,[b,§], Andrea Criscuolo,[a] Daniele Malpicci,[b] Rosa Gaglione,[a] Angela Arciello,[a] Domenica Musumeci,[a,c] Elena Lucenti,[d] Elena Cariati,[b] Daniela Montesarchio,[a,*] and Clelia Giannini[b,*]

Affiliations

[a]*Department of Chemical Sciences, University of Naples Federico II, via Cintia 21, 80126 Napoli, Italy*

[b]*Department of Chemistry, University of Milan, via Golgi 19, 20133 Milano, Italy*

[c]*Institute of Biostructures and Bioimaging (IBB) - CNR, via Tommaso De Amicis 95, 80145 Napoli, Italy*

[d]*Institute of Chemical Sciences and Technologies "Giulio Natta" (SCITEC) of CNR, via Golgi 19, Milano 20133, Italy*

Corresponding author – full address

prof. Daniela Montesarchio, Department of Chemical Sciences, University of Naples Federico II, via Cintia 21, 80126 Napoli, Italy

daniela.montesarchio@unina.it

Compound No.	InChI	Biological Activity on HeLA cancer cells[a] IC_{50} (µM)	Biological Activity on MCF-7 cancer cells[a] IC_{50} (µM)
**TT-(E-2Py)$_2$**	InChI=1S/C23H12N8/c1-3-11-24-17(5-1)7-9-19-15-27-22-29-14-13-26-21(29)30-20(16-28-23(30)31(19)22)10-8-18-6-2-4-12-25-18/h1-6,11-16H	NOT TESTED	NOT TESTED
**TT-(E-2Py)$_3$**	InChI=1S/C30H15N9/c1-4-16-31-22(7-1)10-13-25-19-34-28-37(25)29-35-20-26(14-11-23-8-2-5-	NOT TESTED	NOT TESTED

	17-32-23)39(29)30-36-21-27(38(28)30)15-12-24-9-3-6-18-33-24/h1-9,16-21H		
**TT-(E-4Py)$_2$**	InChI=1S/C23H12N8/c1(17-5-9-24-10-6-17)3-19-15-27-22-29-14-13-26-21(29)30-20(16-28-23(30)31(19)22)4-2-18-7-11-25-12-8-18/h5-16H	NOT TESTED	NOT TESTED
**TT-(E-4Py)$_3$**	InChI=1S/C30H15N9/c1(22-7-13-31-14-8-22)4-25-19-34-28-37(25)29-35-20-26(5-2-23-9-15-32-16-10-23)39(29)30-36-21-27(38(28)30)6-3-24-11-17-33-18-12-24/h7-21H	NOT TESTED	NOT TESTED
**TT-(E-2PyMe$^+$I$^-$)**	InChI=1S/C17H12N7.HI/c1-21-9-3-2-4-13(21)5-6-14-12-20-17-23-10-7-18-15(23)22-11-8-19-16(22)24(14)17;/h2-4,7-12H,1H3;1H/q+1;/p-1	41	>50
**TT-(E-2PyMe$^+$I$^-$)$_2$**	InChI=1S/C25H18N8.2HI/c1-29-14-5-3-7-19(29)9-11-21-17-27-24-31-16-13-26-23(31)32-22(18-28-25(32)33(21)24)12-10-20-8-4-6-15-30(20)2;;/h3-8,13-18H,1-2H3;2*1H/q+2;;/p-2	>50	38
**TT-(E-2PyMe$^+$I$^-$)$_3$**	InChI=1S/C33H24N9.3HI/c1-37-19-7-4-10-25(37)13-16-28-22-34-31-40(28)32-35-23-29(17-14-26-11-5-8-20-38(26)2)42(32)33-36-24-30(41(31)33)18-15-27-12-6-9-21-39(27)3;;;/h4-12,19-24H,1-3H3;3*1H/q+3;;;/p-3	47	29
**TT-(E-4PyMe$^+$I$^-$)**	InChI=1S/C17H12N7.HI/c1-21-8-4-13(5-9-21)2-3-14-12-20-17-23-10-6-18-15(23)22-11-7-19-16(22)24(14)17;/h4-12H,1H3;1H/q+1;/p-1	>50	>50
**TT-(E-4PyMe$^+$I$^-$)$_2$**	InChI=1S/C25H18N8.2HI/c1-29-12-7-19(8-13-29)3-5-21-17-27-24-31-16-11-26-23(31)32-22(18-28-25(32)33(21)24)6-4-20-9-14-30(2)15-10-20;;/h7-18H,1-2H3;2*1H/q+2;;/p-2	>50	>50
**TT-(E-4PyMe$^+$I$^-$)$_3$**	InChI=1S/C33H24N9.3HI/c1-37-16-10-25(11-17-37)4-7-28-22-34-31-40(28)32-35-23-29(8-5-26-12-18-38(2)19-13-26)42(32)33-36-24-30(41(31)33)9-6-27-14-20-39(3)21-15-27;;;/h10-24H,1-3H3;3*1H/q+3;;;/p-3	32	50

[a] Brief description of screening procedure incl. reference.

IC_{50} values were established by testing increasing concentrations of each compound in the 0-50 µM range for 72 h on HeLa and MCF7 cancer cells by MTT assays.
