# Supplementary material for: Combining Cyclic Triimidazo Triazine Core With Ethynyl‐N‐Methyl‐Pyridinium Groups for Targeting G‐Quadruplex Structures
Source: Arch Pharm (Weinheim). 2025 Jul 2;358(7):e70037. doi: 10.1002/ardp.70037 (PMC12223358; doi:10.1002/ardp.70037)
Supplement: Supplementary file 2 — Supp Inf cyclic triimidazoles final revised. [file ARDP-358-e70037-s002.docx]

**Supporting Information**

*to the manuscript:*

**Combining cyclic triimidazo triazine core with ethynyl-*N*-methyl-pyridinium groups for targeting G-quadruplex structures**

**by**

Chiara Platella,^a,§^ Stefano Di Ciolo,^b,§^, Andrea Criscuolo,^a^ Daniele Malpicci,^b^ Rosa Gaglione,^a^ Angela Arciello,^a^ Domenica Musumeci,^a,c^ Elena Lucenti,^d^ Elena Cariati,^b^ Daniela Montesarchio,^a,^* and

Clelia Giannini^b,^*

*^a^Department of Chemical Sciences, University of Naples Federico II, via Cintia 21, 80126 Napoli, Italy*

*^b^Department of Chemistry, University of Milan, via Golgi 19, 20133 Milano, Italy*

*^c^Institute of Biostructures and Bioimaging (IBB) - CNR, via Tommaso De Amicis 95, 80145 Napoli, Italy*

*^d^Institute of Chemical Sciences and Technologies “Giulio Natta” (SCITEC) of CNR, via Golgi 19, Milano 20133, Italy*

*^§^ These authors equally contributed to the work.*

Corresponding authors e-mail: [daniela.montesarchio@unina.it](mailto:daniela.montesarchio@unina.it); [clelia.giannini@unimi.it](mailto:clelia.giannini@unimi.it)

**
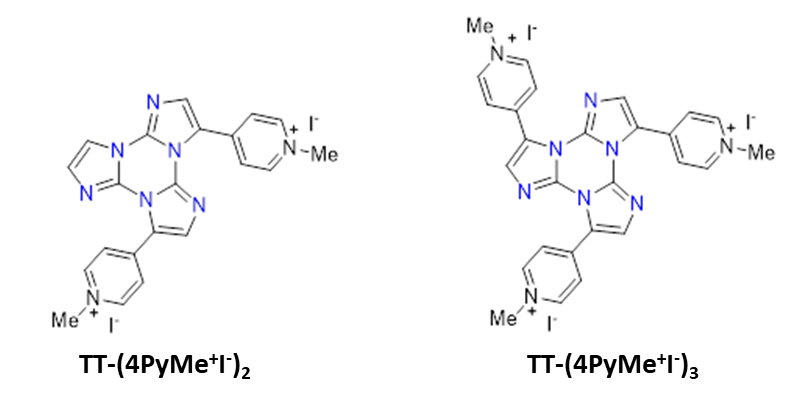
**

**Chart S1.** Chemical structures of **TT-(4PyMe^+^I^-^)_2_** and **TT-(4PyMe^+^I^-^)_3_**.


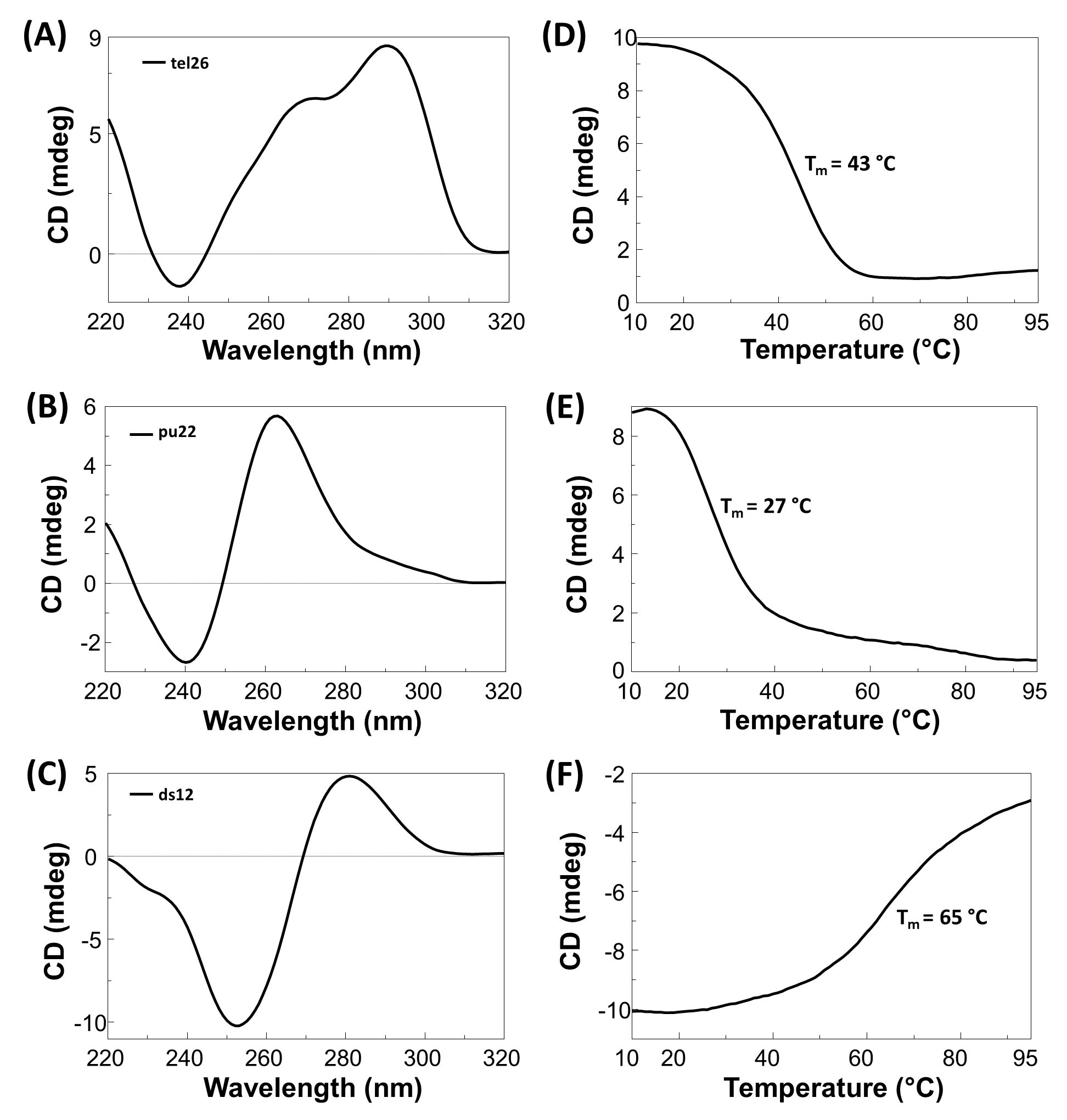


**Figure S1.** Left panels: CD spectra of 2 μM solutions of tel26 (A), pu22 (B) and ds12 (C) in 20 mM KCl, 5 mM potassium phosphate buffer (pH 7) for tel26 and ds12 or in 10 mM Tris-HCl buffer (pH 7) for pu22. Right panels: CD melting curves for tel26 (D), pu22 (E) and ds12 (F) in 20 mM KCl, 5 mM potassium phosphate buffer (pH 7) for tel26 and ds12, recorded at 290 and 253 nm respectively, and in 10 mM Tris-HCl buffer (pH 7) for pu22, recorded at 263 nm.

**
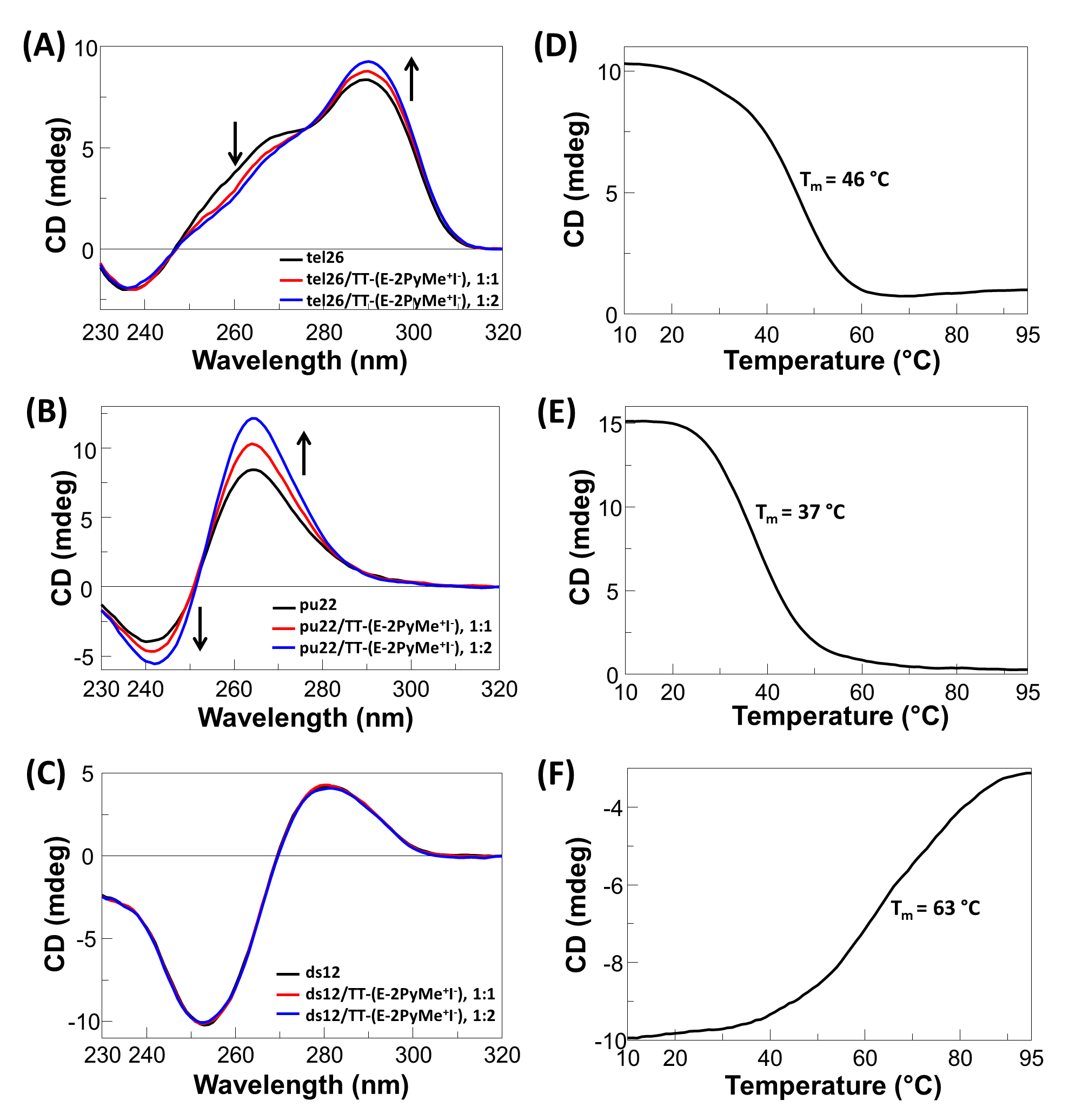
**

**Figure S2.** Left panels: CD spectra of 2 μM solutions of tel26 (A), pu22 (B) and ds12 (C) in 20 mM KCl, 5 mM potassium phosphate buffer (pH 7) for tel26 and ds12 or in 10 mM Tris-HCl buffer (pH 7) for pu22 in the presence of increasing amounts (up to 2 equivalents) of **TT-(E-2PyMe^+^I^-^)**. Arrows indicate the variation of CD bands on increasing ligand concentration. Right panels: CD melting curves for tel26 (D), pu22 (E) and ds12 (F) in the presence of **TT-(E-2PyMe^+^I^-^)** (2 equivalents) in 20 mM KCl, 5 mM potassium phosphate buffer (pH 7) for tel26 and ds12, recorded at 290 and 253 nm respectively, and in 10 mM Tris-HCl buffer (pH 7) for pu22, recorded at 263 nm

**
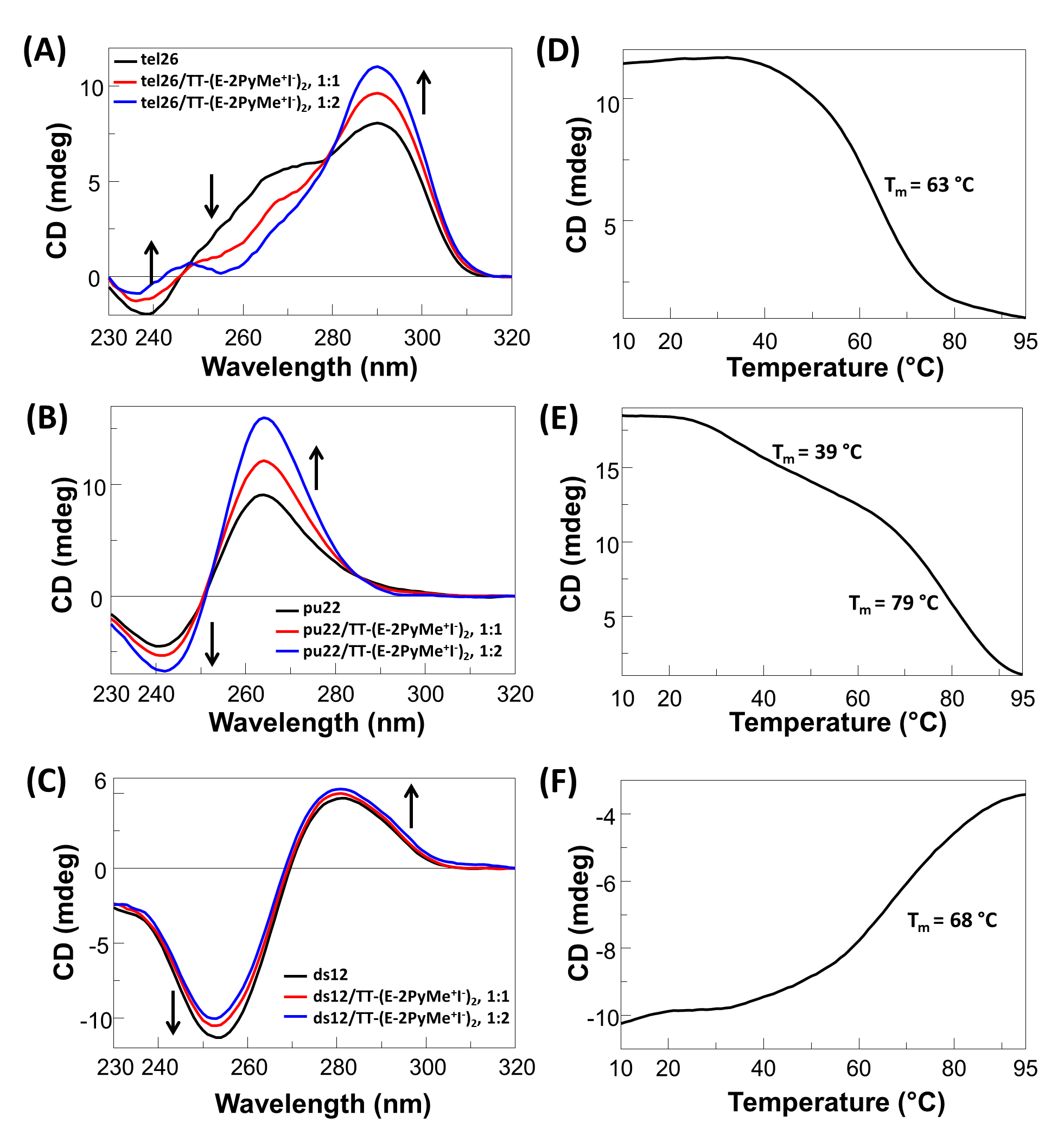
**

**Figure S3.** Left panels: CD spectra of 2 μM solutions of tel26 (A), pu22 (B) and ds12 (C) in 20 mM KCl, 5 mM potassium phosphate buffer (pH 7) for tel26 and ds12 or in 10 mM Tris-HCl buffer (pH 7) for pu22 in the presence of increasing amounts (up to 2 equivalents) of **TT-(E-2PyMe^+^I^-^)_2_**. Arrows indicate the variation of CD bands on increasing ligand concentration. Right panels: CD melting curves for tel26 (D), pu22 (E) and ds12 (F) in the presence of **TT-(E-2PyMe^+^I^-^)_2_** (2 equivalents) in 20 mM KCl, 5 mM potassium phosphate buffer (pH 7) for tel26 and ds12, recorded at 290 and 253 nm respectively, and in 10 mM Tris-HCl buffer (pH 7) for pu22, recorded at 263 nm.

**
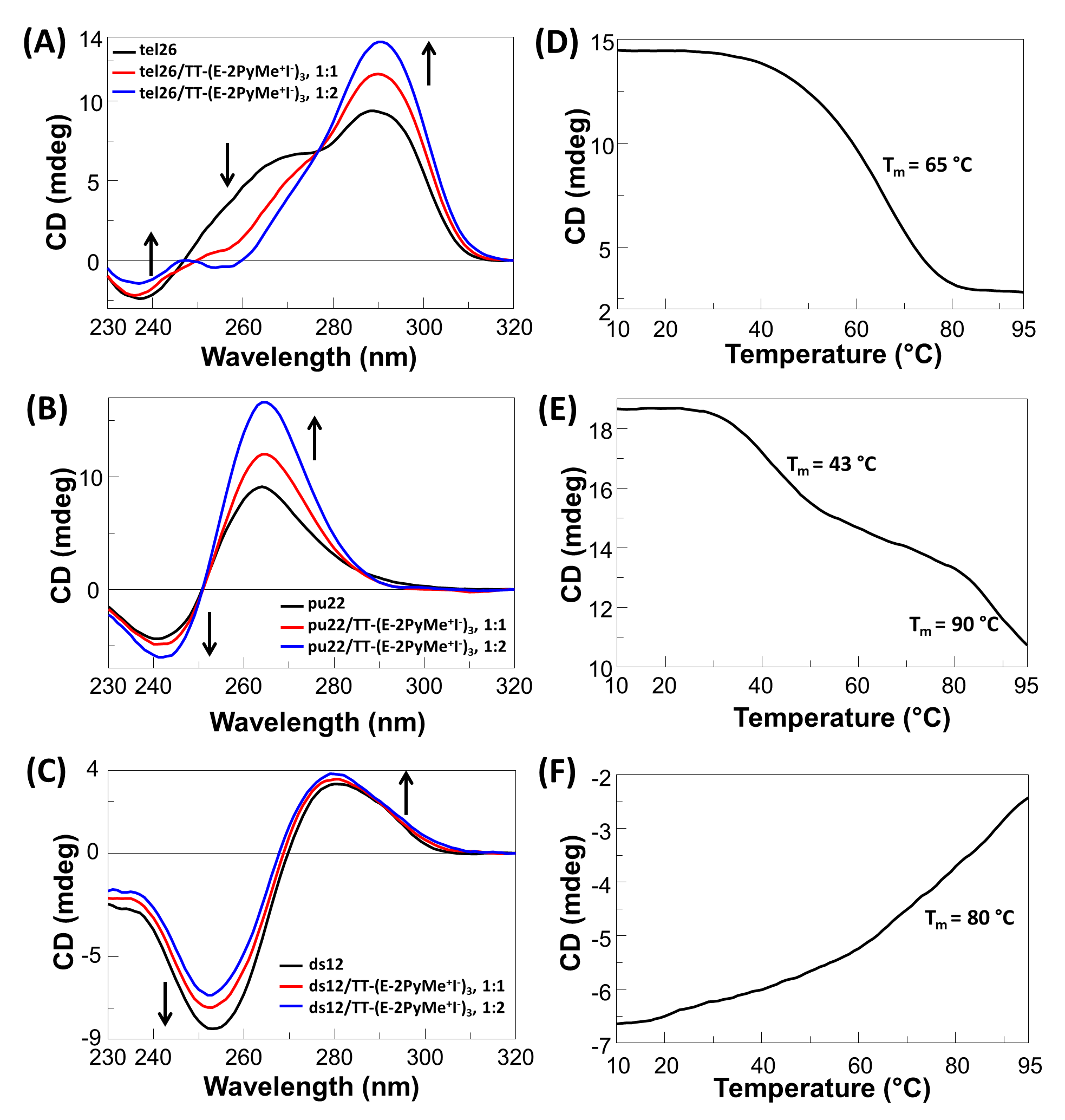
**

**Figure S4.** Left panels: CD spectra of 2 μM solutions of tel26 (A), pu22 (B) and ds12 (C) in 20 mM KCl, 5 mM potassium phosphate buffer (pH 7) for tel26 and ds12 or in 10 mM Tris-HCl buffer (pH 7) for pu22 in the presence of increasing amounts (up to 2 equivalents) of **TT-(E-2PyMe^+^I^-^)_3_**. Arrows indicate the variation of CD bands on increasing ligand concentration. Right panels: CD melting curves for tel26 (D), pu22 (E) and ds12 (F) in the presence of **TT-(E-2PyMe^+^I^-^)_3_** (2 equivalents) in 20 mM KCl, 5 mM potassium phosphate buffer (pH 7) for tel26 and ds12, recorded at 290 and 253 nm respectively, and in 10 mM Tris-HCl buffer (pH 7) for pu22, recorded at 263 nm.

**
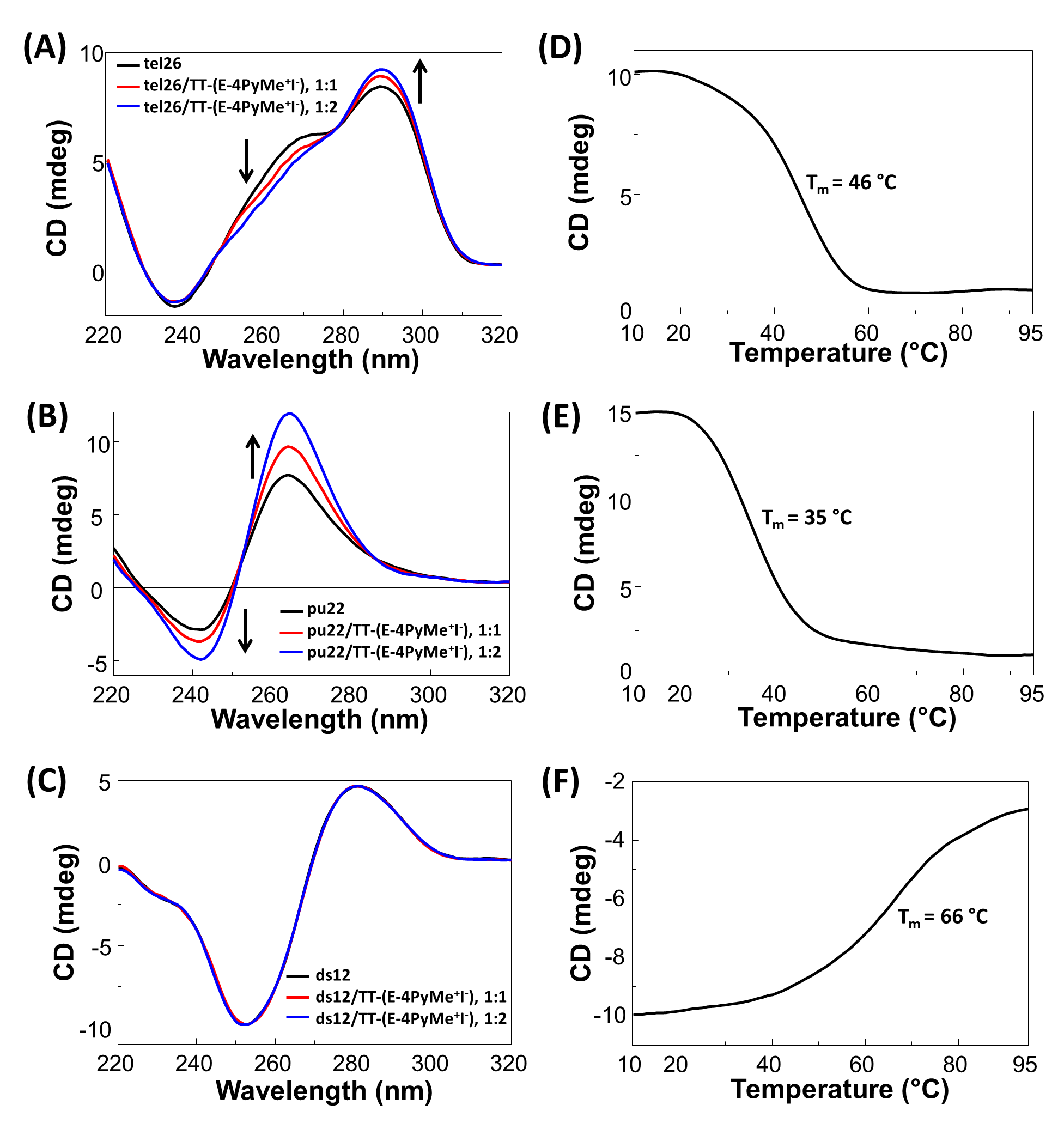
**

**Figure S5.** Left panels: CD spectra of 2 μM solutions of tel26 (A), pu22 (B) and ds12 (C) in 20 mM KCl, 5 mM potassium phosphate buffer (pH 7) for tel26 and ds12 or in 10 mM Tris-HCl buffer (pH 7) for pu22 in the presence of increasing amounts (up to 2 equivalents) of **TT-(E-4PyMe^+^I^-^)**. Arrows indicate the variation of CD bands on increasing ligand concentration. Right panels: CD melting curves for tel26 (D), pu22 (E) and ds12 (F) in the presence of **TT-(E-4PyMe^+^I^-^)** (2 equivalents) in 20 mM KCl, 5 mM potassium phosphate buffer (pH 7) for tel26 and ds12, recorded at 290 and 253 nm respectively, and in 10 mM Tris-HCl buffer (pH 7) for pu22, recorded at 263 nm.

**
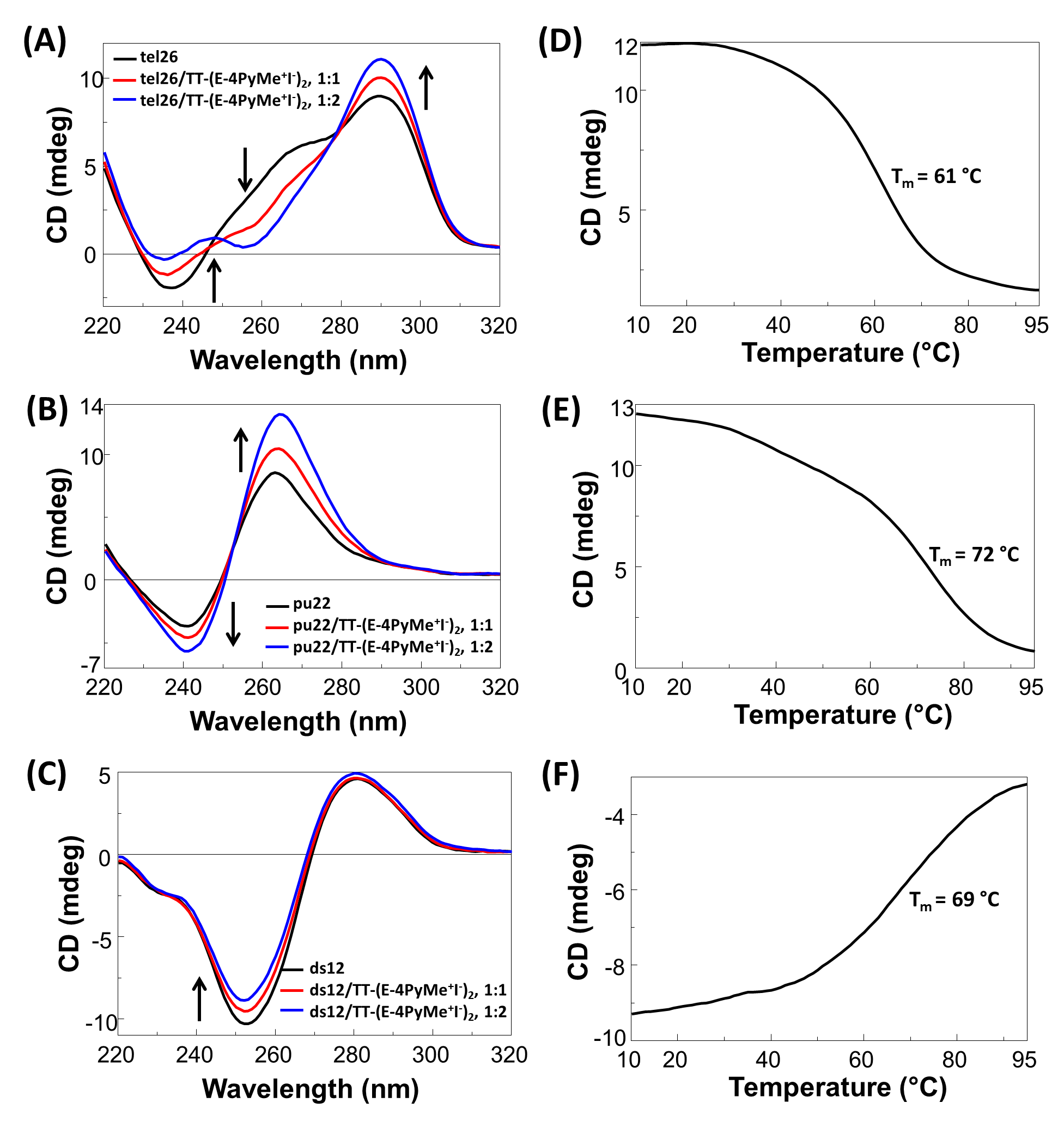
**

**Figure S6.** Left panels: CD spectra of 2 μM solutions of tel26 (A), pu22 (B) and ds12 (C) in 20 mM KCl, 5 mM potassium phosphate buffer (pH 7) for tel26 and ds12 or in 10 mM Tris-HCl buffer (pH 7) for pu22 in the presence of increasing amounts (up to 2 equivalents) of **TT-(E-4PyMe^+^I^-^)_2_**. Right panels: CD melting curves for tel26 (D), pu22 (E) and ds12 (F) in the presence of **TT-(E-4PyMe^+^I^-^)_2_** (2 equivalents) in 20 mM KCl, 5 mM potassium phosphate buffer (pH 7) for tel26 and ds12, recorded at 290 and 253 nm respectively, and in 10 mM Tris-HCl buffer (pH 7) for pu22, recorded at 263 nm.

**
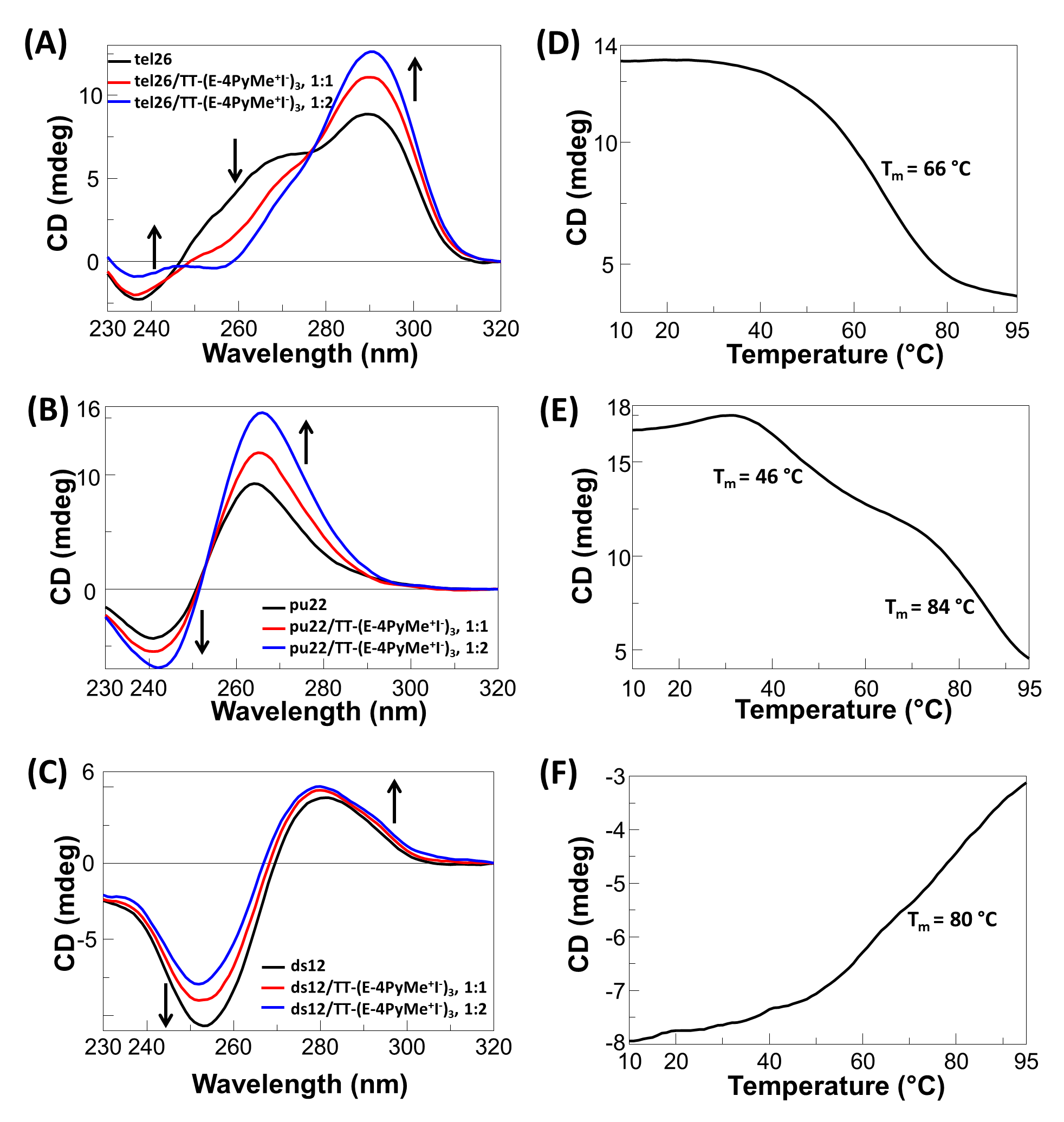
**

**Figure S7.** Left panels: CD spectra of 2 μM solutions of tel26 (A), pu22 (B) and ds12 (C) in 20 mM KCl, 5 mM potassium phosphate buffer (pH 7) for tel26 and ds12 or in 10 mM Tris-HCl buffer (pH 7) for pu22 in the presence of increasing amounts (up to 2 equivalents) of **TT-(E-4PyMe^+^I^-^)_3_**. Right panels: CD melting curves for tel26 (D), pu22 (E) and ds12 (F) in the presence of **TT-(E-4PyMe^+^I^-^)_3_** (2 equivalents) in 20 mM KCl, 5 mM potassium phosphate buffer (pH 7) for tel26 and ds12, recorded at 290 and 253 nm respectively, and in 10 mM Tris-HCl buffer (pH 7) for pu22, recorded at 263 nm.

**
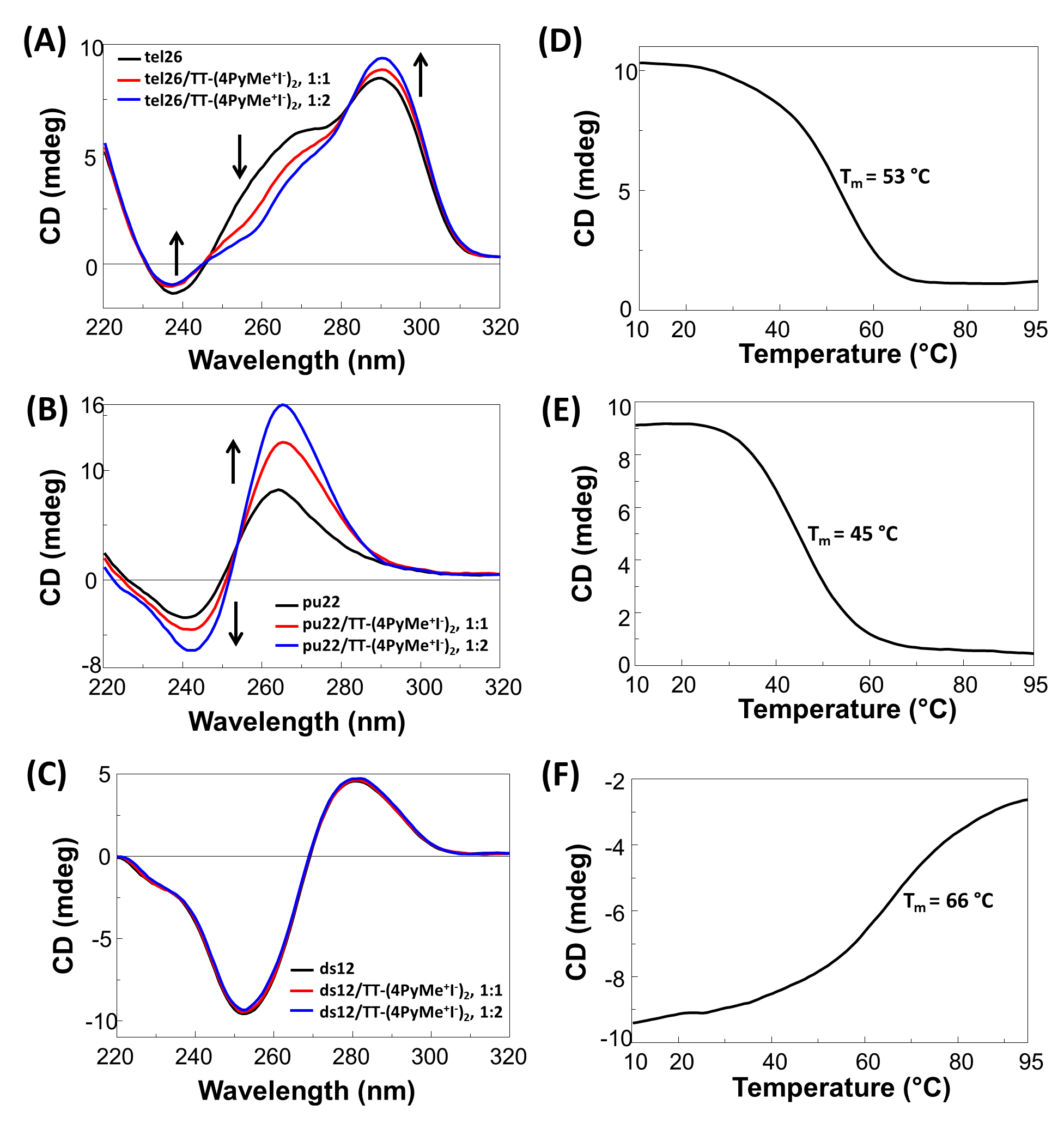
**

**Figure S8.** Left panels: CD spectra of 2 μM solutions of tel26 (A), pu22 (B) and ds12 (C) in 20 mM KCl, 5 mM potassium phosphate buffer (pH 7) for tel26 and ds12 or in 10 mM Tris-HCl buffer (pH 7) for pu22 in the presence of increasing amounts (up to 2 equivalents) of **TT-(4PyMe^+^I^-^)_2_**. Right panels: CD melting curves for tel26 (D), pu22 (E) and ds12 (F) in the presence of **TT-(4PyMe^+^I^-^)_2_** (2 equivalents) in 20 mM KCl, 5 mM potassium phosphate buffer (pH 7) for tel26 and ds12, recorded at 290 and 253 nm respectively, and in 10 mM Tris-HCl buffer (pH 7) for pu22, recorded at 263 nm.

**
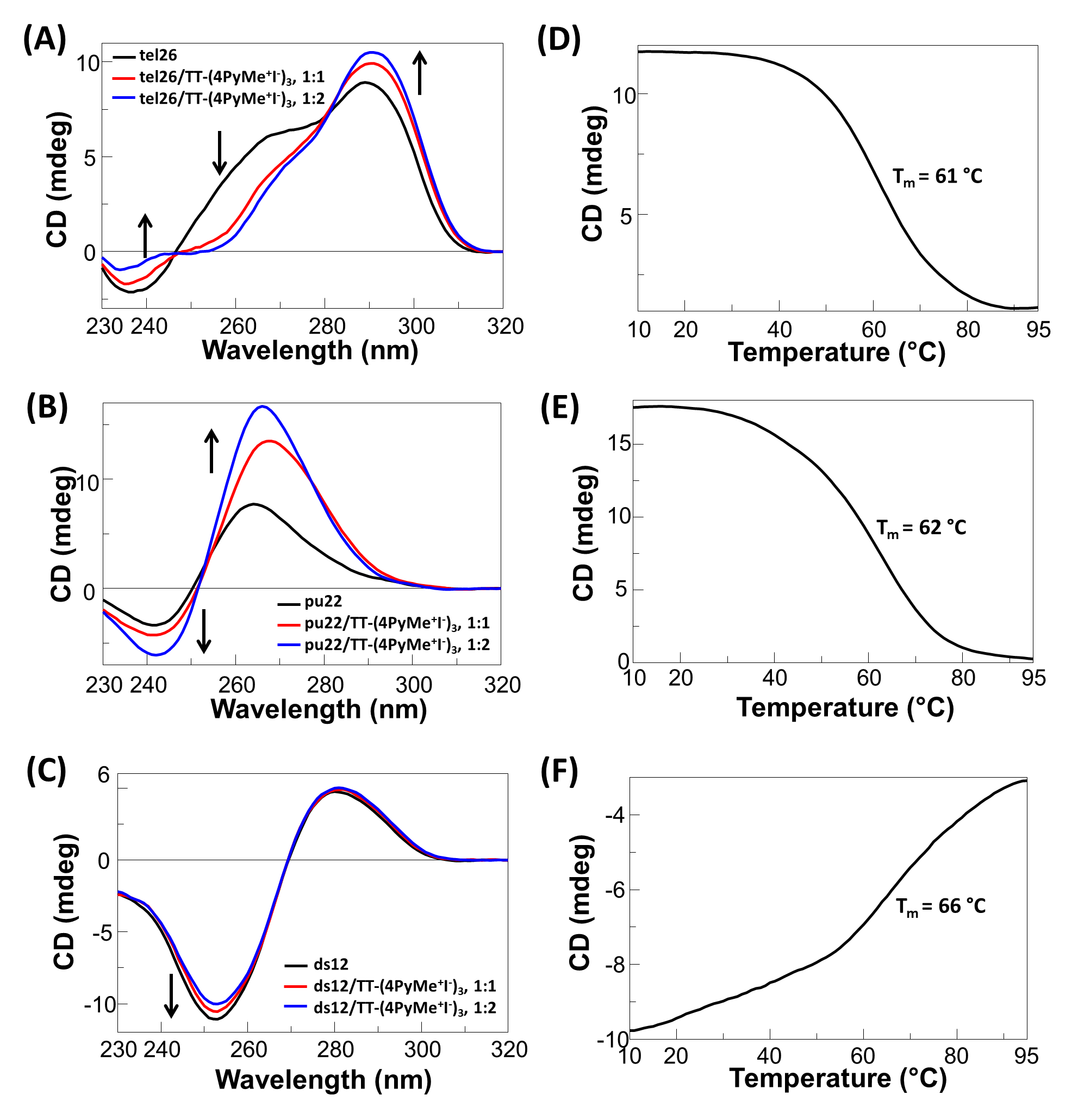
**

**Figure S9.** Left panels: CD spectra of 2 μM solutions of tel26 (A), pu22 (B) and ds12 (C) in 20 mM KCl, 5 mM potassium phosphate buffer (pH 7) for tel26 and ds12 or in 10 mM Tris-HCl buffer (pH 7) for pu22 in the presence of increasing amounts (up to 2 equivalents) of **TT-(4PyMe^+^I^-^)_3_**. Right panels: CD melting curves for tel26 (D), pu22 (E) and ds12 (F) in the presence of **TT-(4PyMe^+^I^-^)_3_** (2 equivalents) in 20 mM KCl, 5 mM potassium phosphate buffer (pH 7) for tel26 and ds12, recorded at 290 and 253 nm respectively, and in 10 mM Tris-HCl buffer (pH 7) for pu22, recorded at 263 nm.

**
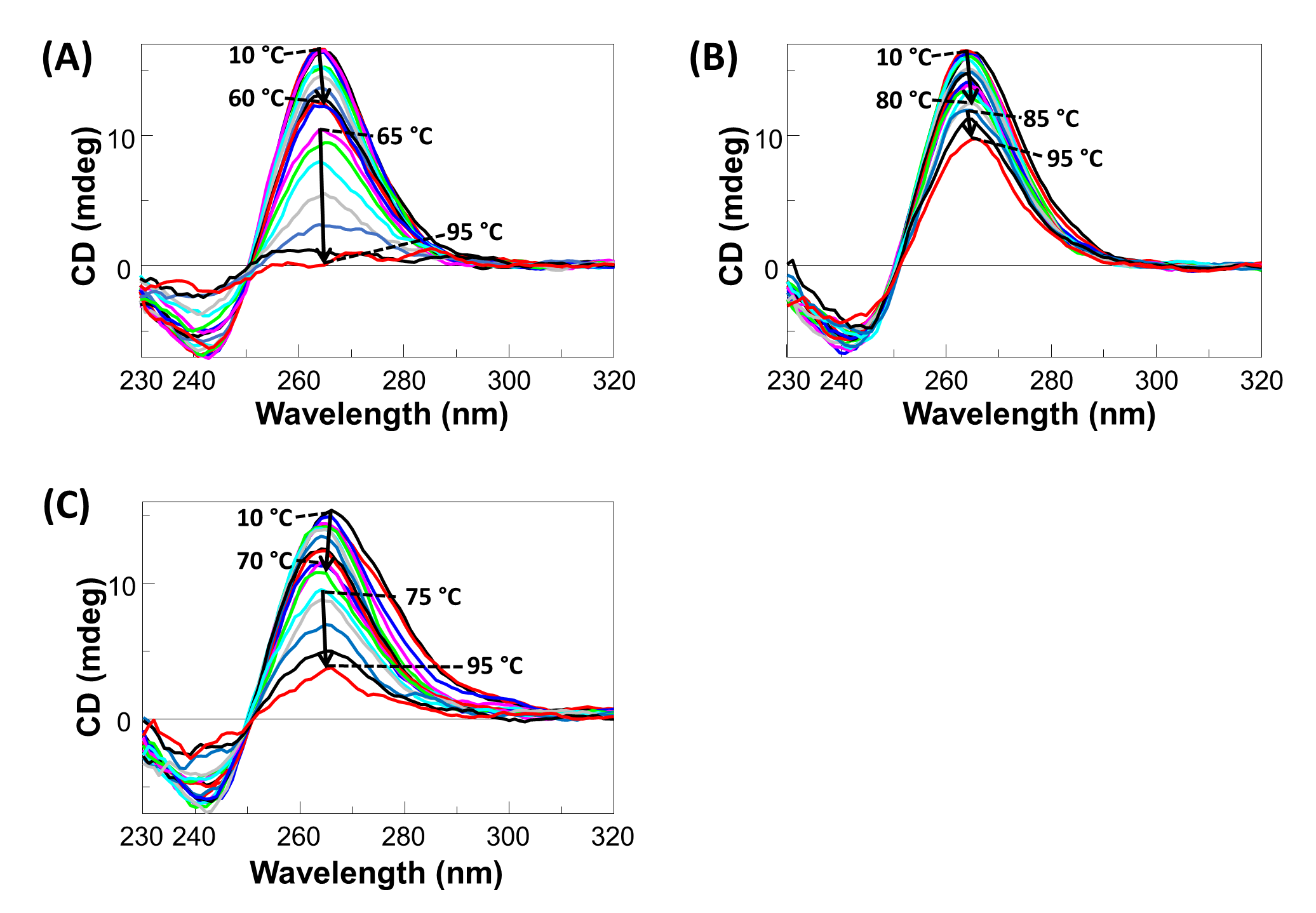
**

**Figure S10.** CD spectra of 2 μM solutions of pu22 in 10 mM Tris-HCl buffer (pH 7) in the presence of 2 equivalents of **TT-(E-2PyMe^+^I^-^)_2_** (A), **TT-(E-2PyMe^+^I^-^)_3_** (B) and **TT-(E-4PyMe^+^I^-^)_3_** (C) at different temperatures. Arrows indicate the variation of CD bands on increasing the temperature from 10 °C to 95 °C.

**
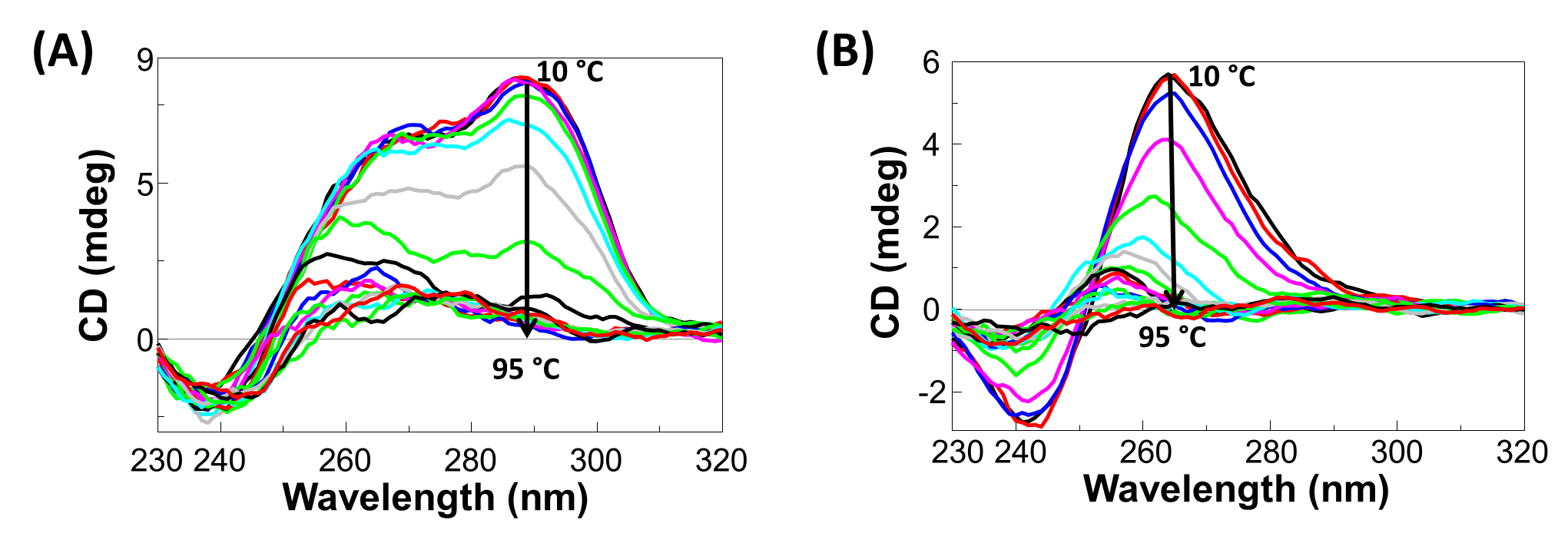
**

**Figure S11.** CD spectra of 2 μM solutions of tel26 (A) in 20 mM KCl, 5 mM potassium phosphate buffer (pH 7) and pu22 (B) in 10 mM Tris-HCl buffer (pH 7) at different temperatures. Arrows indicate the variation of CD bands on increasing the temperature from 10 °C to 95 °C.


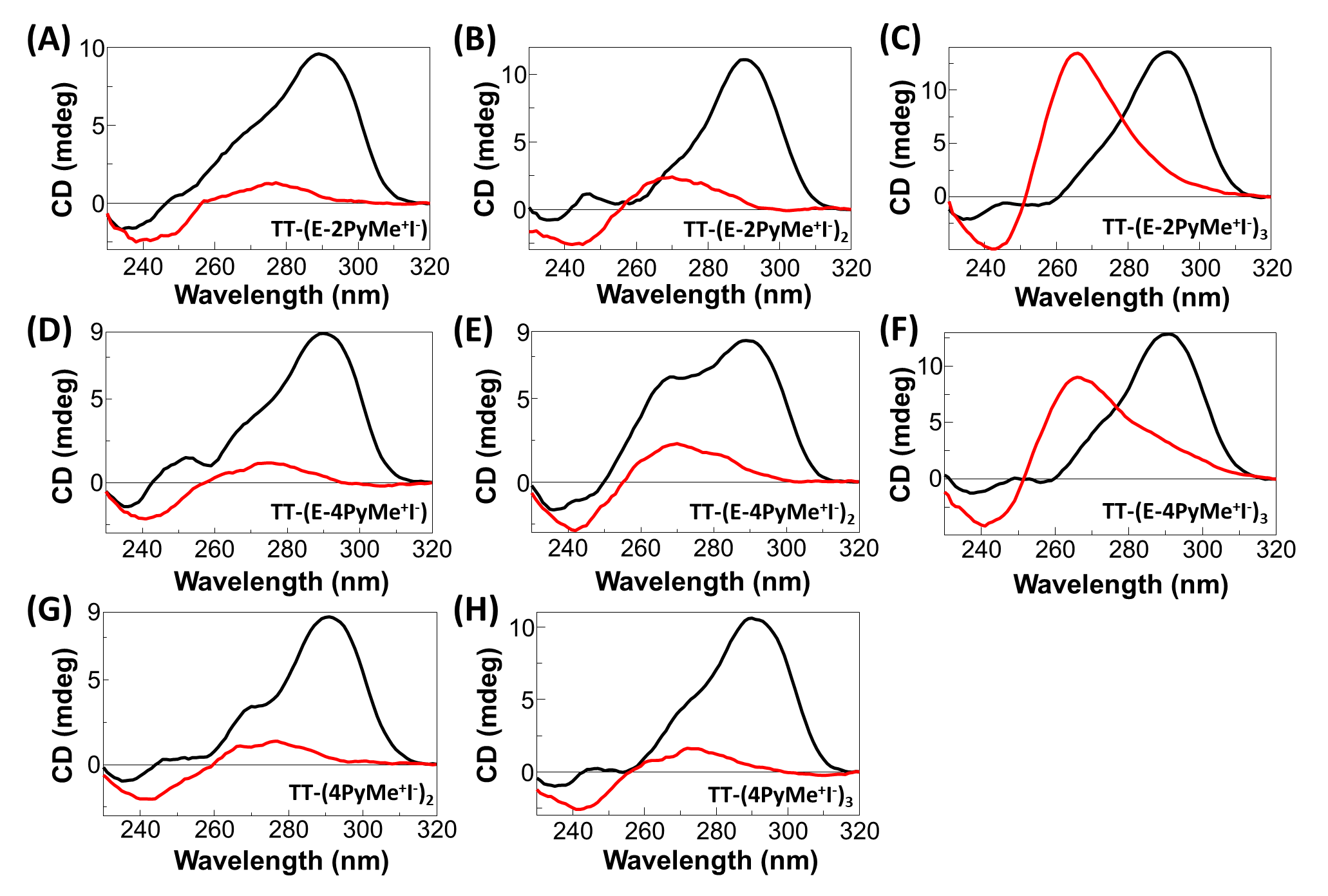


**Figure S12.** CD spectra of 2 μM solutions of tel26 in 20 mM KCl, 5 mM potassium phosphate buffer (pH 7) in the presence of 2 equivalents of **TT-(E-2PyMe^+^I^-^)** (A), **TT-(E-2PyMe^+^I^-^)_2_** (B), **TT-(E-2PyMe^+^I^-^)_3_** (C), **TT-(E-4PyMe^+^I^-^)** (D), **TT-(E-4PyMe^+^I^-^)_2_** (E), **TT-(E-4PyMe^+^I^-^)_3_** (F), **TT-(4PyMe^+^I^-^)_2_** (G) and **TT-(4PyMe^+^I^-^)_3_** (H) at 10 °C (black curves) and 95 °C (red curves).


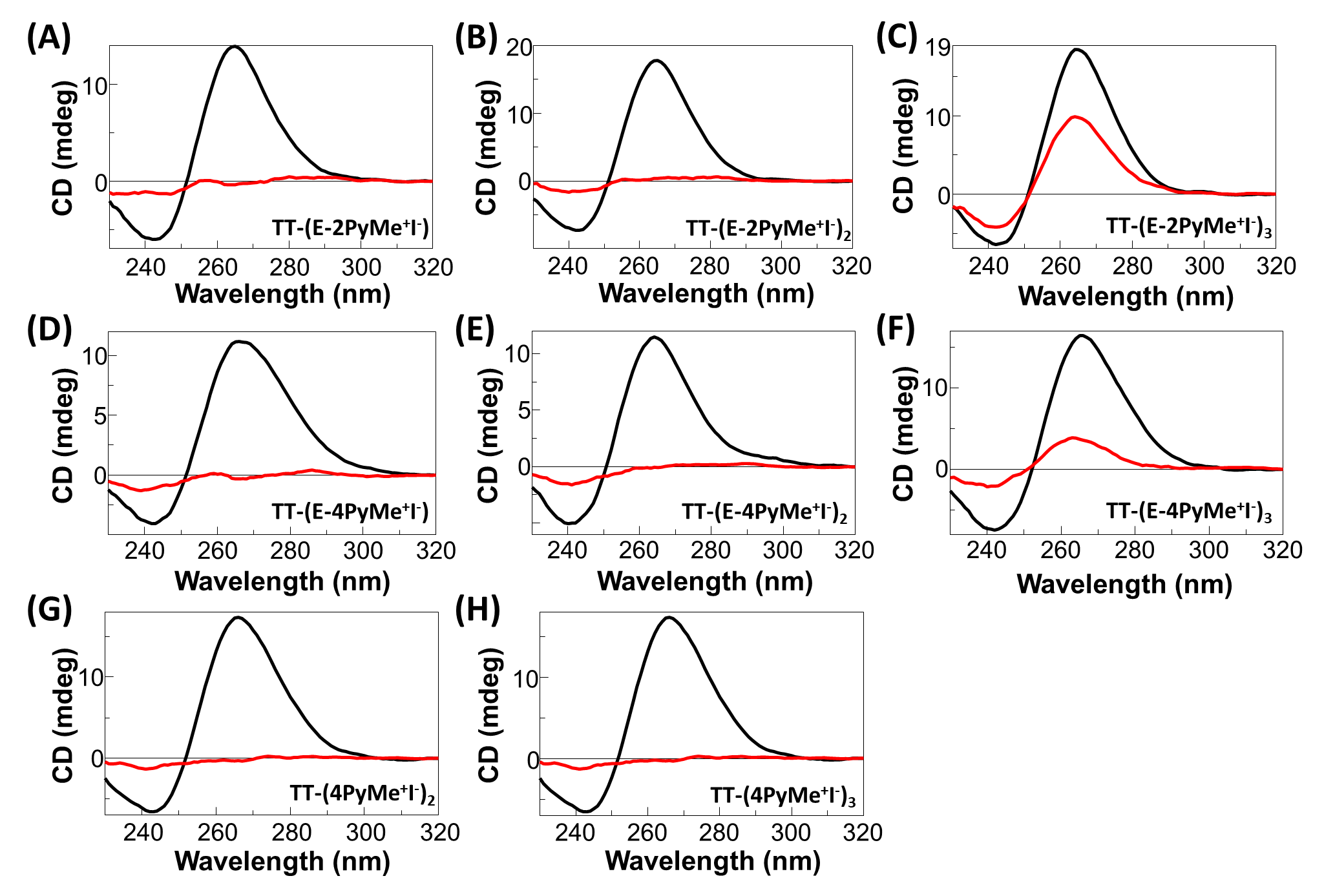


**Figure S13.** CD spectra of 2 μM solutions of pu22 in 10 mM Tris-HCl buffer (pH 7) in the presence of 2 equivalents of **TT-(E-2PyMe^+^I^-^)** (A), **TT-(E-2PyMe^+^I^-^)_2_** (B), **TT-(E-2PyMe^+^I^-^)_3_** (C), **TT-(E-4PyMe^+^I^-^)** (D), **TT-(E-4PyMe^+^I^-^)_2_** (E), **TT-(E-4PyMe^+^I^-^)_3_** (F), **TT-(4PyMe^+^I^-^)_2_** (G) and **TT-(4PyMe^+^I^-^)_3_** (H) at 10 °C (black curves) and 95 °C (red curves).

**
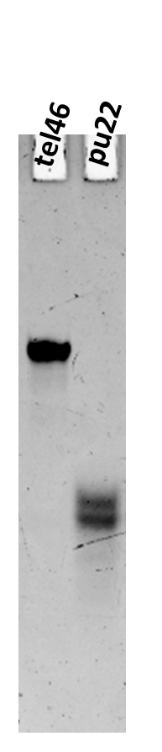
**

**Figure S14.** Native PAGE experiment: tel46 and pu22 samples were loaded at 1 and 2 μM concentration, respectively, in 20 mM KCl, 5 mM potassium phosphate buffer (pH 7) and 10 mM Tris-HCl buffer (pH 7), respectively, and analyzed by 20% native PAGE using Tris-Borate-EDTA (TBE 1x) as running buffer (2.5 h, 100 V, r.t.). Gels were visualized by GelGreen™ staining.

**
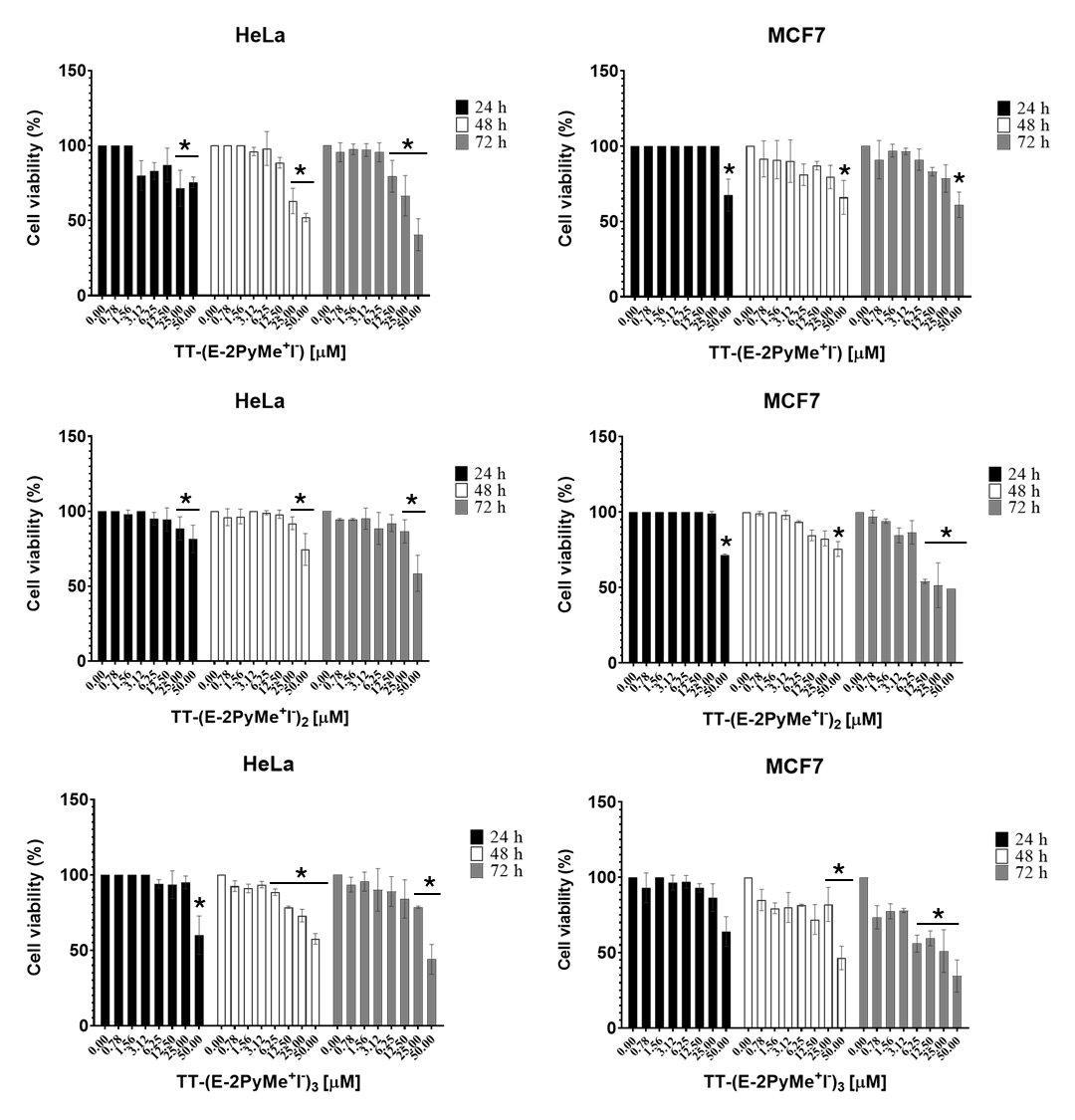
**

**Figure S15.** Effects of increasing concentrations (0-50 μM) of **TT-(E-2PyMe^+^I^-^)**, **TT-(E-2PyMe^+^I^-^)_2_** and **TT-(E-2PyMe^+^I^-^)_3_** on the viability of HeLa and MCF7 human cells after 24, 48 and 72 h of incubation. Cell viability values are reported as the percentage of viable treated cells with respect to control cells grown in the absence of the tested compounds. Three independent experiments were performed with triplicated determinations. *P ≤ 0.05 was obtained for treated *vs.* control samples.

**
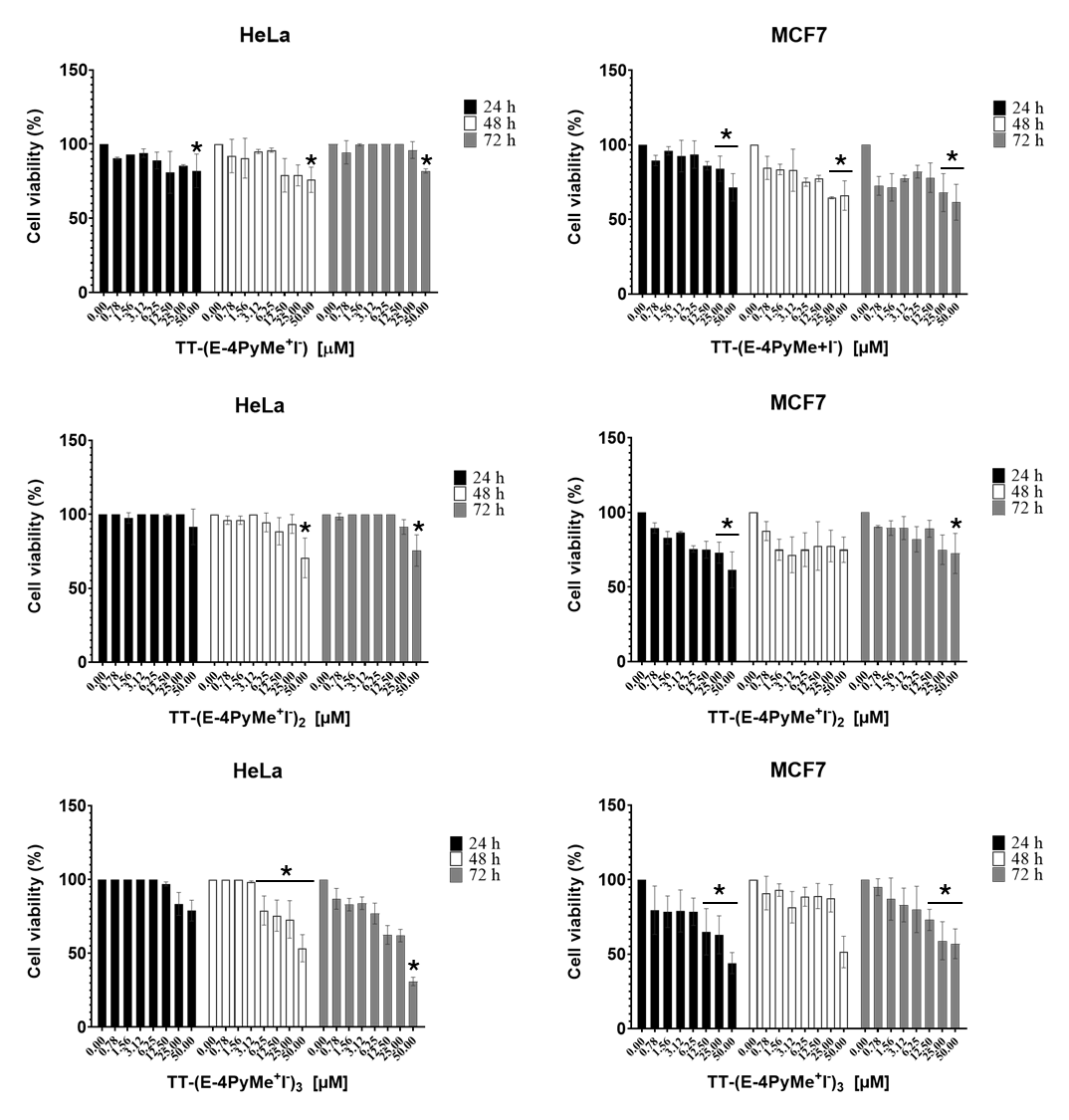
**

**Figure S16.** Effects of increasing concentrations (0-50 μM) of **TT-(E-4PyMe^+^I^-^)**, **TT-(E-4PyMe^+^I^-^)_2_** and **TT-(E-4PyMe^+^I^-^)_3_** on the viability of HeLa and MCF7 human cells after 24, 48 and 72 h of incubation. Cell viability values are reported as the percentage of viable treated cells with respect to control cells grown in the absence of the tested compounds. Three independent experiments were performed with triplicated determinations. *P ≤ 0.05 was obtained for treated *vs.* control samples.

**
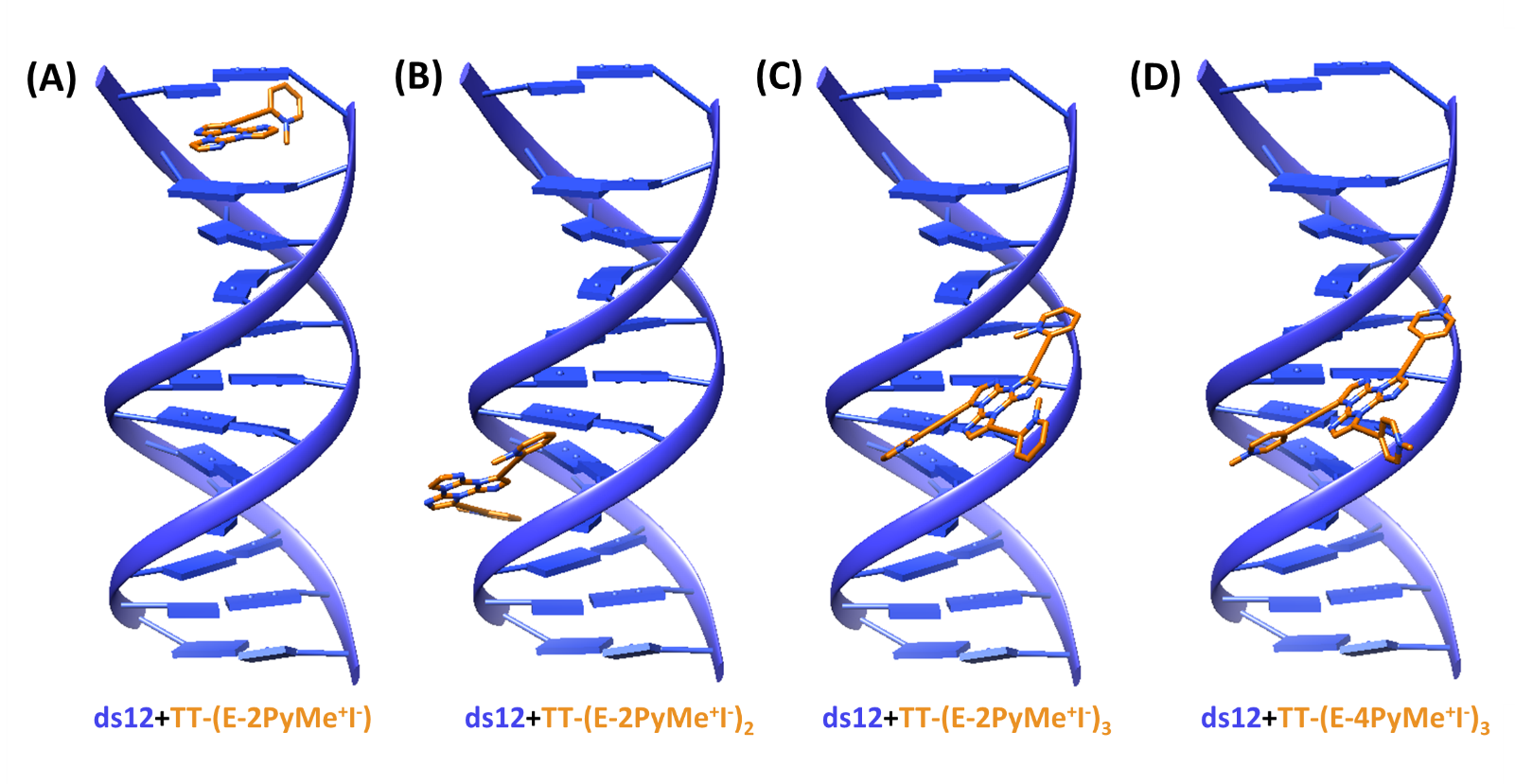
**

**Figure S17.** Binding modes of **TT-(E-2PyMe^+^I^-^)**, **TT-(E-2PyMe^+^I^-^)_2_**, **TT-(E-2PyMe^+^I^-^)_3_**, **TT-(E-4PyMe^+^I^-^)_3_** when docked to the ds12 duplex prepared from PDB 1NAJ (A, B, C, D, respectively). Ligands and duplexes are represented as orange sticks and blue ribbon, respectively.

**Table S1.** Binding energies calculated by AutoDock Vina for **TT-(E-2PyMe^+^I^-^)**, **TT-(E-2PyMe^+^I^-^)_2_**, **TT-(E-2PyMe^+^I^-^)_3_**, **TT-(E-4PyMe^+^I^-^)_3_** when docked to tel26 G-quadruplex (PDB 5MVB), pu22 G-quadruplex (PDB 2L7V) and ds12 duplex (prepared from PDB 1NAJ).

|  | Binding energy (kcal/mol) | | |
| --- | --- | --- | --- |
|  | **tel26**  (PDB 5MVB) | **pu22**  (PDB 2L7V) | **ds12**  (PDB prepared from 1NAJ) |
| **TT-(E-2PyMe^+^I^-^)** | -8.6 | -8.2 | -8.1 |
| **TT-(E-2PyMe^+^I^-^)_2_** | -9.2 | -8.3 | -9.2 |
| **TT-(E-2PyMe^+^I^-^)_3_** | -9.9 | -9.3 | -9.4 |
| **TT-(E-4PyMe^+^I^-^)_3_** | -9.6 | -8.8 | -9.6 |

**Figure S18. HPLC-MS** profile of **TT-E-2Py**

**Figure S19. ^1^H-NMR (CD_2_Cl_2_, 400 MHz)** of **TT-E-2Py**

**Figure S20. ^13^C-NMR (CD_2_Cl_2_, 100 MHz)** of **TT-E-2Py**

**Figure S21. HPLC-MS** profile of **TT-(E-2Py)_2_**

**Figure S22. ^1^H-NMR (CD_2_Cl_2_, 400 MHz)** of **TT-(E-2Py)_2_**

**Figure S23. ^13^C-NMR (CD_2_Cl_2_, 100 MHz)** of **TT-(E-2Py)_2_**

**Figure S24. HPLC-MS** profile of **TT-(E-2Py)_3_**

**Figure S25. ^1^H-NMR (CD_2_Cl_2_, 400 MHz)** of **TT-(E-2Py)_3_**

**Figure S26. ^13^C-NMR (CD_2_Cl_2_, 100 MHz)** of **TT-(E-2Py)_3_**

**Figure S27. HPLC-MS** profile of **TT-(E-4Py)**

**Figure S28. ^1^H-NMR (CD_2_Cl_2_, 400 MHz)** of **TT-(E-4Py)**

**Figure S29. ^13^C-NMR (CD_2_Cl_2_, 100 MHz)** of **TT-(E-4Py)**

**Figure S30. HPLC-MS** profile of **TT-(E-4Py)_2_**

**Figure S31. ^1^H-NMR (CD_2_Cl_2_, 400 MHz)** of **TT-(E-4Py)_2_**

**Figure S32. ^13^C-NMR (CD_2_Cl_2_, 100 MHz)** of **TT-(E-4Py)_2_**

**Figure S33. HPLC-MS** profile of **TT-(E-4Py)_3_**

**Figure S34. ^1^H-NMR (CD_2_Cl_2_, 400 MHz)** of **TT-(E-4Py)_3_**

**Figure S35. ^13^C-NMR (CD_2_Cl_2_, 100 MHz)** of **TT-(E-4Py)_3_**

**Figure S36. HPLC-MS** profile of **TT-E-2PyMe^+^I^-^**

**Figure S37. ^1^H-NMR (D_2_O, 400 MHz)** of **TT-E-2PyMe^+^I^-^**

with water signal suppression

**Figure S38. ^13^C-NMR (D_2_O, 100 MHz)** of **TT-E-2PyMe^+^I^-^**

**Figure S39. HPLC-MS** profile of **TT-(E-2PyMe^+^I^-^)_2_**

**Figure S40. ^1^H-NMR (DMSO, 400 MHz)** of **TT-(E-2PyMe^+^I^-^)_2_**

**Figure S41. ^13^C-NMR (DMSO, 100 MHz)** of **TT-(E-2PyMe^+^I^-^)_2_**

**Figure S42. HPLC-MS** profile of **TT-(E-2PyMe^+^I^-^)_3_**

**Figure S43. ^1^H-NMR (DMSO, 400 MHz)** of **TT-(E-2PyMe^+^I^-^)_3_**

**Figure S44. ^13^C-NMR (DMSO, 100 MHz)** of **TT-(E-2PyMe^+^I^-^)_3_**

**Figure S45. HMBC spectrum** of **TT-(E-2PyMe^+^I^-^)_3_**

**Figure S46. HPLC-MS** profile of **TT-(E-4PyMe^+^I^-^)**

**Figure S47. ^1^H-NMR (DMSO, 400 MHz)** of **TT-(E-4PyMe^+^I^-^)**

**Figure S48. ^13^C-NMR (D_2_O, 100 MHz)** of **TT-(E-2PyMe^+^I^-^)**

**Figure S49. HPLC-MS** profile of **TT-(E-4PyMe^+^I^-^)_2_**

**Figure S50. ^1^H-NMR (D_2_O, 400 MHz)** of **TT-(E-4PyMe^+^I^-^)_2_**

**Figure S51. ^13^C-NMR (D_2_O, 100 MHz)** of **TT-(E-4PyMe^+^I^-^)_2_**

**Figure S52. HPLC-MS** profile of **TT-(E-4PyMe^+^I^-^)_3_**

**Figure S53. ^1^H-NMR (DMSO, 400 MHz)** of **TT-(E-4PyMe^+^I^-^)_3_**

**Figure S54. ^13^C-NMR (DMSO, 100 MHz)** of **TT-(E-4PyMe^+^I^-^)_3_**

**
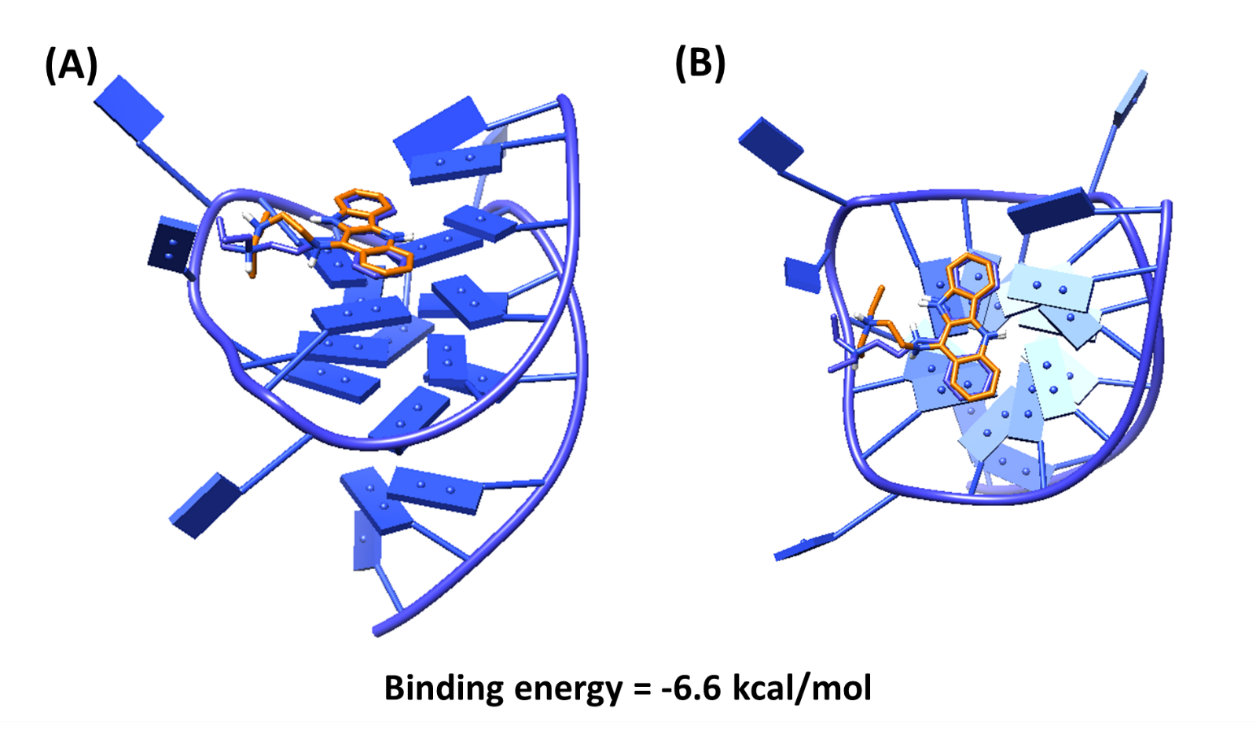
**

**Figure S55.** Comparison between the binding mode of Quindoline in the NMR structure (blue) and Quindoline when docked to the pu22 G-quadruplex PDB 2L7V (orange). Side view (A) and top view (B). Ligand and G-quadruplex are represented as blue/orange sticks and blue ribbon, respectively.
